# Antennal transcriptome analysis of the Asian longhorned beetle *Anoplophora glabripennis*

**DOI:** 10.1038/srep26652

**Published:** 2016-05-25

**Authors:** Ping Hu, Jingzhen Wang, Mingming Cui, Jing Tao, Youqing Luo

**Affiliations:** 1 Key Laboratory for Silviculture and Conservation of Ministry of Education, Beijing Forestry University, Beijing, 100083, P. R. China

## Abstract

Olfactory proteins form the basis of insect olfactory recognition, which is crucial for host identification, mating, and oviposition. Using transcriptome analysis of *Anoplophora glabripennis* antenna, we identified 42 odorant-binding proteins (OBPs), 12 chemosensory proteins (CSPs), 14 pheromone-degrading enzymes (PDEs), 1 odorant-degrading enzymes (ODE), 37 odorant receptors (ORs), 11 gustatory receptors (GRs), 2 sensory neuron membrane proteins (SNMPs), and 4 ionotropic receptor (IR). All CSPs and PBPs were expressed in antennae, confirming the authenticity of the transcriptome data. CSP expression profiles showed that *Agla*CSP3, *Agla*CSP6, and *Agla*CSP12 were expressed preferentially in maxillary palps and *Agla*CSP7 and *Agla*CSP9 were strongly expressed in antennae. The vast majority of CSPs were highly expressed in multiple chemosensory tissues, suggesting their participation in olfactory recognition in almost all olfactory tissues. Intriguingly, the PBP *AglaPBP2* was preferentially expressed in antenna, indicating that it is the main protein involved in efficient and sensitive pheromone recognition. Phylogenetic analysis of olfactory proteins indicated *Agla*GR1 may detect CO_2_. This study establishes a foundation for determining the chemoreception molecular mechanisms of *A. glabripennis*, which would provide a new perspective for controlling pest populations, especially those of borers.

During the coevolution of plants and insects, communication systems evolved that rely strongly on the insect olfactory system. For instance, feeding, orientation, the identification of suitable hosts, mating, and oviposition are necessary survival processes that depend on the olfactory system. The Asian longhorned beetle (ALB) *Anoplophora glabripennis* (Motschulsky) (Coleoptera: Cerambycidae) has been documented in 31 host genera and is recognized as one of the most damaging invasive insects in Europe and North America^1^,^2^, where it causes considerable economic losses, as well as in its native region in Asia.

Multiple stratagems that are currently used to control ALB can be classified into two types. One, based on an ecological perspective, attempts to control *A. glabripennis* in China by planting mixtures of sensitive and protected tree species. Both types are planted together to identify host trees and resistant or rarely infested trees. Sensitive trees are used as a bait to attract ALB, enabling insects to be centralized and killed, and ultimately controls the insect population. This results in the protection of non-preferred trees to some extent^3^. Sjöman *et al.* found that 36 species in 31 genera are at risk of attack, while 31 species in 16 genera are resistant or rarely infested^2^. The second type has relied on the identification of ALB pheromones and plant volatiles. This has provided the basis for artificial insect trapping, which has greatly benefited eradication and management programs in both introduced and native ranges. To date, three compounds have been identified as ALB male pheromones: 4-(*n*-heptyloxy)butan-1-ol, 4-(*n*-heptyloxy)butanal^4^, and (3E,6E)-α-farnesene^5^. For the sesquiterpene (3E, 6E)-α-farnesene, both male and female beetles are antennal responsive. Further studies are required to understand fully the mechanism of odorant detection in insects, including how chemicals are sensed by antennae and the associated reactions.

With the application of pheromone and plant volatile traps, research has focused on the olfactory system. Many sensillum are located in insect antennae. Volatile molecules move through pores on the sensillum to olfactory sensory neurons (OSNs) located in the lymph^6^. Three main types of proteins are involved in peri-receptor and receptor events in olfactory sensilla: those that bind odorants, including odorant-binding proteins (OBPs) and chemosensory proteins (CSPs); transmembrane proteins, including odorant receptors (ORs), ionotropic receptors (IRs), gustatory receptors (GRs), and sensory neuron membrane proteins (SNMPs); and enzymes that degrade odorants, including pheromone-degrading enzymes (PDEs) and odorant-degrading enzymes (ODEs)^7^. These proteins are involved in the olfactory pathway for odorant sensing and interact with each other to form a unified, functional olfactory system.

These proteins have been characterized in recent studies. Binding proteins bind hydrophobic odorants through the pores of sensilla and transport them through the sensilla lymph to facilitate their solubilization. Pheromone-binding proteins (PBPs) are a special type of OBP that specifically bind to pheromone compounds via five α-helices, forming a combined cavity. In fact, some PBPs bind to specific pheromones, such as PBP1 and PBP2 of *Antheraea pernyi* (Guérin-Méneville)^8^. Other PBPs cannot distinguish between pheromones and non-pheromones, e.g., SlitPBP of *Spodoptera litura* (Fabricius)^9^. Evidence for PBP-binding specificity to a single pheromone component remains elusive^10^. OBPs have been studied in detail with respect to their structure, binding ability, expression orientation, gland release role, and binding mechanism, particularly in Lepidoptera. Among other binding proteins, chemosensory proteins (CSPs) have fewer cysteine residues (4) and are smaller than OBPs, and bind far more odorants than OBPs^11^. Their locations are not limited to chemosensory organs; accordingly, they probably have other functions in addition to transporting odorants^12^.

Membrane proteins are located in the outer dendrites of ORNs and are involved in odorant reception and signal transduction, or bind to volatile chemicals^13^. Furthermore, insect ORs form heteromeric complexes with a highly conserved and universal co-receptor, Orco^14^. The co-receptor constitutes a ligand-gated nonselective cation channel and binds specific ligands via negatively charged amino acid residues, forming selective ionic pore checkpoints. These have been examined in *Xenopus laevis* oocytes using the voltage-clamp technique for *Bmor*OR1 and *Bmor*OR3^15^. Intriguingly, new odorant sensory elements that were identified using Sf21 cell lines stably expressing *Bmor*OR1 and *Bmor*OR3, the Orco family gene *Bmor*Orco, and GCaMP3, which are sensitive at ppb levels of odorant chemicals in solution, can selectively distinguish between similar odorant chemical structures owing to the selectivity of the highly conserved and insect-specific Orco and odorant receptors^16^. The identification of the co-receptor has led to the development of new insect control and monitoring methods.

IRs are a conserved family of synaptic ligand-gated ion channels that evolved from ionotropic glutamate receptors (iGluRs) and have similar structures, including an extracellular N-terminus, a bipartite ligand-binding domain, two lobes separated by an ion channel domain, and a short cytoplasmic C-terminal region^17^. IRs are essential for odor-evoked neuronal response neurons; mutations in IR84a, IR64a, IR8a, and IR25a of *Drosophila* inhibit odor-evoked neuronal responses^18^,^19^. A key property of IRs is their localization to the dendrites of OSNs housed in ceoloconic sensilla^20^. However, the specificity by which IRs recognize their ligands is unclear^17^. Some members of the IR superfamily are expressed in taste neurons^21^. Intriguingly, *Drosophila* IR94b has been implicated in auditory system functions^22^.

GRs are generally expressed in gustatory receptor neurons in gustatory organs, which combine soluble tastants or pheromones^23^,^24^, and transport gustatory signals. However, some GRs are expressed in antennal dendrites and respond to carbon dioxide^25^,^26^. Concretely, in *Drosophila melanogaster* where GRs have been mostly studied, *DmelDmel*GR5a^27^, *Dmel*GR64a^28^, and *Dmel*GR64f^29^ showed a response to sugars; *Dmel*GR43a^30^ acted as a specific fructose receptor; *Dmel*GR 33a^31^ detected a wide range of bitter tasting chemicals; and *Dmel*GR 21a and *Dmel*GR 63a^32^ detected carbon dioxide.

SNMPs belong to the CD36 protein family, which participate in pheromone recognition^33^. There are two types of SNMPs, SNMP1 and SNMP2. Both are expressed in the sensillum trichodeum, but they differ in location of expression, and level of expression. Benton *et al.* proposed that SNMP1 acts as docking protein, decreases the stability of PBP and ligand complexes, promotes ligand dissociation, and interacts with ORs in *Drosophila melanogaster*^34^. However, a paper published by Jin *et al.* suggests that SNMPs can act directly on ORs^35^. When SNMP1 is knocked out in *D. melanogaster*, pheromone recognition ability is lost and the ability to sense normal odors is affected. These observations demonstrated that SNMPs are crucial for pheromone detection.

The degradation of odors after they activate receptors and signal termination is enforced by the degradation of ODEs and PDEs. A few PDEs have been identified to date in the coleopteran species *Phyllopertha diversa*^36^ and *Popillia japonica*^37^.

In Coleoptera, most olfactory proteins were identified in 12 species using genomic and transcriptomic analyses. In Lepidoptera, olfactory proteins of large number of species were identified by antennal transcriptome or genome; besides transcriptomes of the ovipositor gland, such as that of *Chilo suppressalis*, revealed 31 chemoreception genes^38^. The olfactory proteins of Coleoptera have not been much less well studied than those of Lepidoptera, the largest order. Olfactory recognition mechanisms of insects differ depending on the insect’s environment and life history, highlighting the need for additional studies in Coleoptera.

In this study, we examined the expression of various olfactory proteins in *A. glabripennis*. This work lays the foundation for studies of the olfactory system and may facilitate the classification of olfactory receptive mechanisms, providing a theoretical basis for new pest control methods that impede the main olfactory recognition processes.

## Results

### Transcriptome sequencing and sequence assembly

To assemble the transcriptome sequence, we generated approximately 33 million raw reads. The Q20 and Q30 base call accuracies for reads were 94.80% and 88.31%, respectively. Assembly resulted in 18,336 unigenes, with an N50 of 1,523 bp. The largest unigene was 8,597 bp. The clean reads for *A. glabripennis* were deposited in the NCBI SRA database [GenBank: SRR2682325]. We found that 66.61% of unigenes matched entries in the NCBI non-redundant (nr) protein database using blastx with a cut-off E-value of 1e^−5^. Moreover, we observed the highest percentage of sequences matches to loci in *T. castaneum* (70.26%), followed by *Acyrthosiphon pisum* (2.85%) and *D. ponderosae* (1.61%). The remaining 25.28% of sequences showed similarity with the sequences of other insects.

### Gene ontology annotation

**Figure 1:**
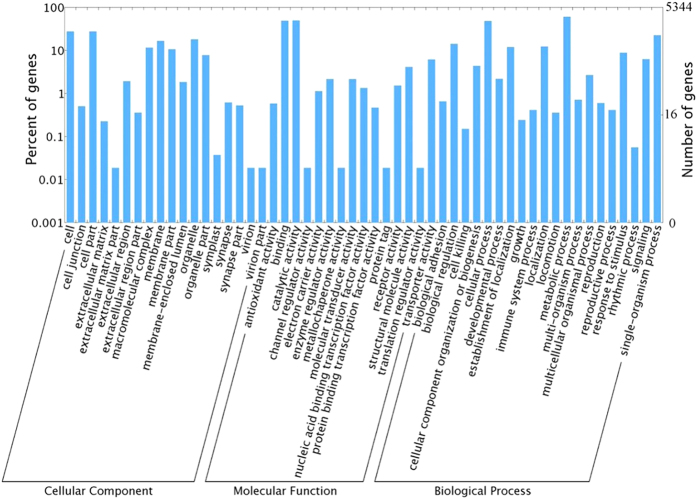
Gene Ontology (GO) results. GO analysis of 5344 genes in *Anoplophora glabripennis,* according to their involvement in biological processes, molecular function, or role as a cellular component.

We used gene ontology (GO) to classify the 5344 genes into functional groups using BLAST2GO. In the ALB transcriptome, molecular function accounted for the majority of GO annotations (82.33%), followed by biological process (67.2%) and cellular components (40.77%). In the molecular function category, antioxidant activity, binding, and transporter activity were the most highly represented terms. In the biological process category, cellular component organization, biogenesis, metabolic process, and single-organism process were the most abundant terms. For the cellular component category, cell, cell part, and organelle were the most abundant terms (Fig. 1).

### Nonreceptor olfactory gene families

#### Odorant-binding proteins

**Figure 2:**
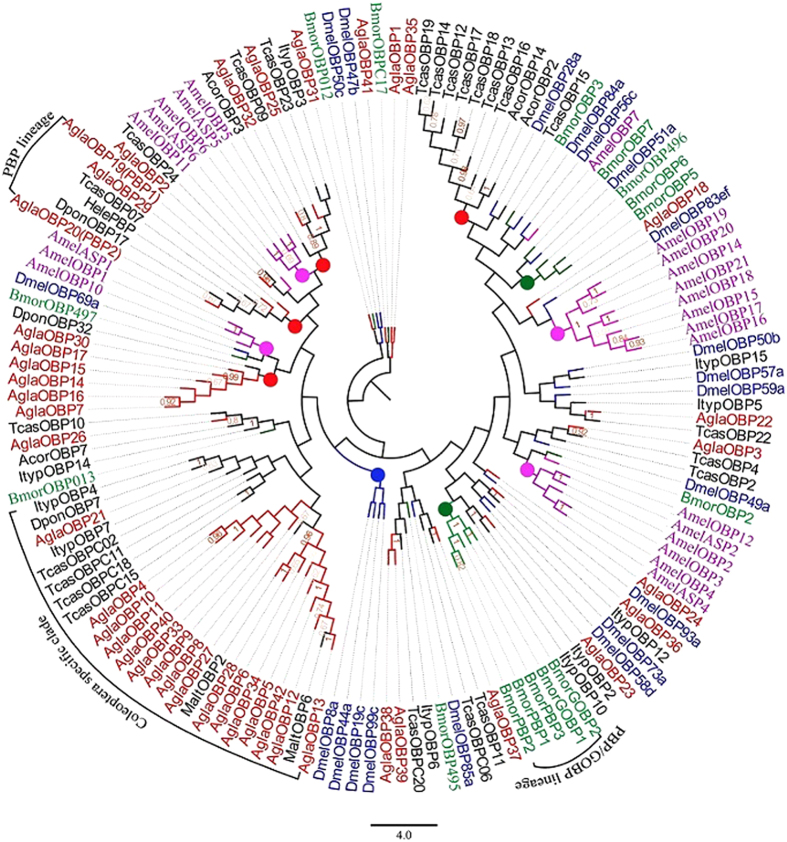
Neighbor-joining phylogenetic tree of candidate odorant-binding proteins (OBPs). The NJ phylogenetic analysis of OBPs of *Anoplophora glabripennis* (*Agla*OBP, red) was performed with reference OBPs of *Monochamus alternatus* (MaltOBP, dark), *Anomala corpulenta* (AcorOBP, dark), *Dendroctonus ponderosae* (*Dpon*OBP, dark), *Ips typographus* (*Ityp*OBP, dark), *Hylamorpha elegans* (*Hele*PBP, dark), *Tribolium castaneum* (*Tcas*OBP, dark), *Drosophila melanogaster* (*Dmel*OBP, Diptera, blue), *Bombyx mori* (*Bmor*OBP, Lepidoptera, green)*, and Apis mellifera* (*Amel*OBP, Hymenoptera, purple). The stability of the nodes was assessed by bootstrap analysis with 1,000 replications, and only bootstrap values ≥0.6 are shown at the corresponding nodes. The scale bar represents 4.0 substitutions per site.

We identified a total of 42 transcripts encoding putative OBPs in *A.* g*labripennis;* twenty-three were full-length genes encoding signal peptides and complete open reading frames (ORFs) longer than 400 bp. According to the OBP classification system, 17 were minus-C class, which lack two cysteine residues (C2 and C5), and two (*Alga19* and *Alga20*) were PBPs (Table 1). Remarkably, we observed multiple unigenes that exhibited blast hits with the same accession number for a particular species. Specifically, *OBP38* and *OBP39* both matched odorant binding protein C20 [GenBank: EFA01425.1]; *OBP19* and *OBP20* matched pheromone binding protein PBP2 [GenBank: AIV43009.1] in *B. horsfieldi*; *OBP30* and *OBP31* showed similarity with odorant binding protein 1 [GenBank: ABR53888.1]; *OBP33*, *OBP34*, *OBP27*, and *OBP28* matched odorant-binding protein 2 [GenBank: AHA39267.1]; *OBP14*, *OBP15*, *OBP16*, and *OBP17* matched minus-C odorant binding protein 2 [GenBank: ADD70031.1]; and *OBP4*, *OBP12*, *OBP13*, *OBP1*, and *OBP5* matched minus-C odorant binding protein 3 [GenBank: ADD82416.1] in *M. alternatus.* Additionally, *OBP6*, *OBP7*, *OBP8*, *OBP9*, *OBP10*, and *OBP11* matched minus-C odorant binding protein 4 [GenBank: ADD82417.1] in *B. horsfieldi* (see supplementary Table S1).In the phylogenetic tree (Fig. 2), we observed only one Coleoptera taxon-specific clade, which contained 16 OBPs of *A.* g*labripennis* (*Agla*OBP21, *AglaAgla*OBP13, *AglaAgla*OBP12, *AglaAgla*cOBP5, *Agla*OBP42, *Agla*OBP27, *Agla*OBP6, *Agla*OBP28, *Agla*OBP34, *Agla*OBP9, *Agla*OBP33, *Agla*OBP8, *Agla*OBP40, *Agla*OBP10, *Agla*OBP4, and *Agla*OBP11) and nine OBPs of other species of Coleoptera. Four Coleoptera lineages formed a tree (red circle), in which one was the Coleoptera specific PBP lineage. Another PBP/GOBP lineage was formed by PBPs and GOBPs of Lepidoptera (*Bombyx mori*) (green circles). We also observed another Lepidoptera lineage in the phylogenetic tree (green circle). Additionally, one Diptera (*D. melanogaster*) species-specific lineage (blue circle) and four Hymenoptera (*Apis mellifera*) species-specific lineages (four purple circles) formed a tree. We also detected low amino acid sequence identity among *Agla*OBPs, which ranged from 3.3% to 55%, with an average of 9.15%.

#### Chemosensory proteins

We identified 12 transcripts encoding putative CSPs, in which seven full-length CSPs genes had ORFs exceeding 400 bp, a signal peptide, and four conserved cysteine residues. Notably, all CSPs transcripts with complete ORFs, except for *CSP4*, had a signal peptide. Furthermore, we detected more than four cysteine residues in transcripts encoding putative CSPs (Table 1). In the neighbor-joining tree (see supplementary Figure S1), one Coleoptera specific clade was constructed with six CSPs of *A.* g*labripennis* and 12 CSPs of other Coleoptera species. Another two Coleoptera specific lineages are labelled with red circles. Additionally, one Diptera specific clade contained all CSPs of *D. melanogaste*. We observed two Lepidoptera (*Bombyx mori*) specific lineages (labelled green round) but no Hymenoptera (*Apis mellifera*) species-specific lineage. In addition, we also observed low amino acid sequence identity among *Agla*CSPs, which ranged from 8.9% to 71.8%, with an average of 11.06%.

#### Pheromone-degrading enzymes

**Figure 3:**
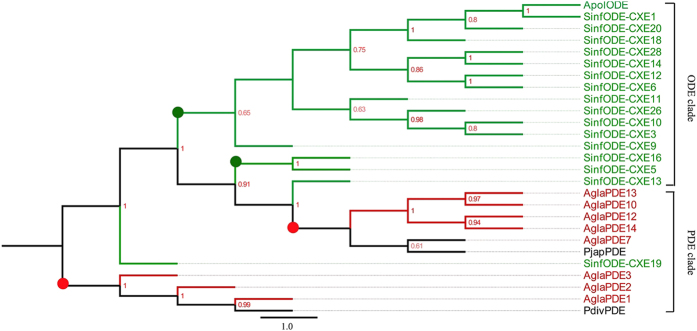
Neighbor-joining tree of candidate pheromone-degrading enzymes (PDEs). The NJ phylogenetic analysis of PDEs of *A. glabripennis*(*Agla*PDE, red) was performed with reference PDEs of *Phyllopertha diversa* (*Pdiv*PDE, dark), *Popillia japonica* (*Pjap*PDE, dark), *Sesamia inferens* (*Sinf*ODE, Lepidoptera, green) and *Antheraea polyphemus* (*Apol*ODE, green). The stability of the nodes was assessed by bootstrap analysis with 1,000 replications, and only bootstrap values ≥0.6 are shown at the corresponding nodes. The scale bar represents 1.0 substitution per site.

We detected 14 transcripts encoding putative PDEs and one transcript encoding a putative ODE in the *A.* g*labripennis* antennal transcriptome. Notably, putative PDEs formed two groups. *Agla*PDE1-*Agla*PDE6 exhibited best blast matches with pheromone-degrading enzyme [GenBank: AAT38512.1] of *Phyllopertha diversa*. *Agla*PDE7–*Agla*PDE14 had best blast matches with pheromone-degrading enzyme [GenBank: AAX58713.5] of *Popillia japonica*. Additionally, *Agla*ODE1 had a best blast hit with pheromone-degrading enzyme [GenBank: AII21987.1] of *Sesamia inferens* (see supplementary Table S2). In the phylogenetic analysis of PDEs (Fig. 3), we observed two Coleoptera-specific clades and two Lepidoptera specific clades (red circle and green circle, respectively). The Coleoptera-specific clades contained all PDEs and ODEs of all Coleoptera species. Additionally, two Lepidoptera-specific clades, except for SinODE-CXE19, included all ODEs of Lepidoptea species. Intriguingly, all PDEs clustered with PDEs consistently. Except for SinODE-CXE19 and SinODE-CXE13, all ODEs also clustered with ODEs. We detected low amino acid sequence identity among *Agla*PDEs, which ranged from 4.5% to 90.9%, with an average of 23.21%.

#### Sensory neuron membrane proteins

**Figure 4:**
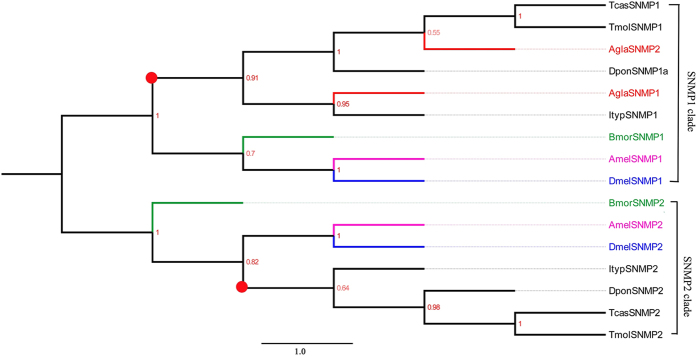
Neighbor-joining tree of candidate sensory neuron membrane proteins (SNMPs). The NJ phylogenetic analysis of SNMPs of *A. glabripennis* (*Agla*SNMP, red) was performed with reference SNMPs of *D. ponderosae* (*Dpon*SNMP, dark), *I. typographus* (*Ityp*SNMP, dark), *T. molitor* (TmolSNMP, dark), *T. castaneum* (*Tcas*SNMP, dark), *D. melanogaster* (*Dmel*SNMP, Diptera, blue), *Bombyx mori* (*Bmor*SNMP, Lepidoptera, green), and *Apis mellifera* (*Amel*SNMP, Hymenoptera, purple). The stability of the nodes was assessed by bootstrap analysis with 1,000 replications, and only bootstrap values ≥0.6 are shown at the corresponding nodes. The scale bar represents 1.0 substitution per site.

We identified two transcripts encoding putative SNMPs. Both had ORFs that were nearly those of full-length genes (exceeded 1,000 bp in size) (see supplementary Table S5). The two *Agla*SNMPs were clustered into a single Coleoptera-specific clade (red circle) in the phylogenetic tree (Fig. 4). In addition, we observed another Coleoptera-specific clade (red circle). Notably, all SNMPs clustered into two clades, SNMP1 and SNMP2. We also detected a mean amino acid sequence identity among *Agla*SNMPs of 9.47%.

### Receptor-encoding genes

#### Odorant receptors

We identified transcripts encoding 37 putative ORs; seven were likely full-length genes that encoded proteins of more than 384 amino acids. Remarkably, four genes (*Agla*OR36*, Agla*OR35*, Agla*OR30, and *Agla*OR19) had best blast matches with odorant receptor 61 [GenBank: EEZ99416.1] of *T. castaneum* (see supplementary Table S3). In the phylogenetic tree (see supplementary Figure S3), most ORs clustered into multiple lineages. One special case was the odorant receptor coreceptor (Orco) lineage, which contained *Bmor*Orco and five Coleoptera Orco (*Tcas*Orco, AcorOrco, DponOrco, McarOrco and TmolOrco), *Agla*OR9, *Amel*OR2, and *Bmor*OR2. Besides, six Coleoptera specific lineages (red circles), three Diptera specific lineages (blue circles), seven Lepidoptera-specific lineages (green circles) and one Hymenoptera specific lineages (purple circle) were present in the neighbor-joining tree. In addition, we detected low amino acid sequence identity among *Agla*ORs, ranging from 2.1% to 77.1%, with an average of 7.74%.

#### Ionotropic receptors

**Figure 5:**
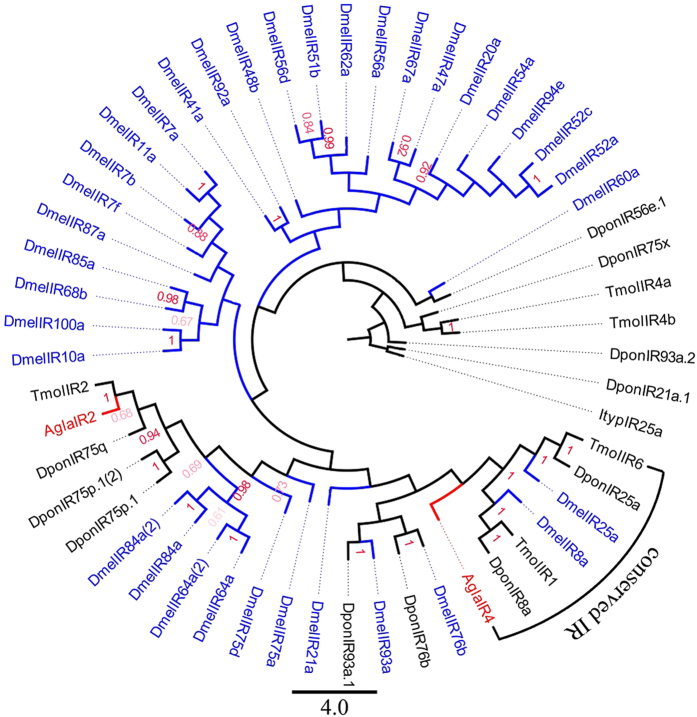
Neighbor-joining phylogenetic tree of candidate ionotropic receptor s (IRs). The NJ phylogenetic analysis of IRs of *A. glabripennis* (*Agla*GR, red) was performed with reference IRs of *D. ponderosae* (*Dpon*IR, dark), *I. typographus* (*Ityp*IR, dark), *T. castaneum* (*Tcas*IR, dark), *Drosophila melanogaster* (*Dmel*IR, Diptera, blue), *Bombyx mori* (*Bmor*IR, Lepidoptera, green), and *Apis mellifera* (*Amel*IR, Hymenoptera, purple). The stability of the nodes was assessed by bootstrap analysis with 1,000 replications, and only bootstrap values ≥0.6 are shown at the corresponding nodes. The scale bar represents 4.0 substitution per site.

We identified four IRs. The ORF sequence lengths of *Agla*IR2 and *Agla*IR4 were long (more than 1300 bp), indicating that they were intact full-length unigenes (see supplementary Table S4). The conserved co-receptor groups IR25a and IR8a formed a phylogenetic tree of IRs, which contained *Agla*IR4 (Fig. 5).

#### Gustatory receptors

**Figure 6:**
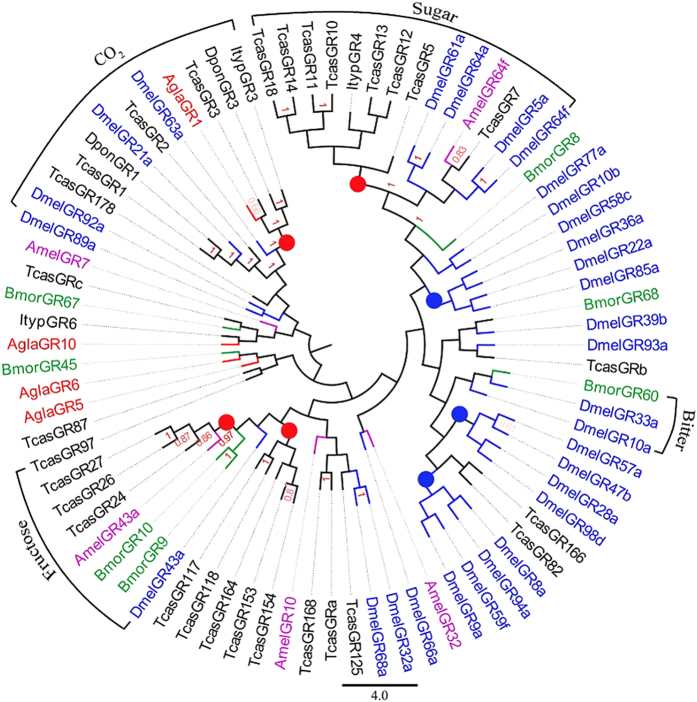
Neighbor-joining phylogenetic tree of candidate gustatory receptors (GRs). The NJ phylogenetic analysis of GRs of *A. glabripennis* (*Agla*GR, red) was performed with reference GRs of *D. ponderosae* (*Dpon*GR, dark), *I. typographus* (*Ityp*GR, dark), *T. castaneum* (*Tcas*GR, dark), *Drosophila melanogaster* (*Dmel*GR, Diptera, blue), *Bombyx mori* (*Bmor*GR, Lepidoptera, green), and *Apis mellifera* (*Amel*GR, Hymenoptera, purple). The stability of the nodes was assessed by bootstrap analysis with 1,000 replications, and only bootstrap values ≥0.6 are shown at the corresponding nodes. The scale bar represents 4.0 substitution per site.

Using a bioinformatics approach, we identified 11 transcripts encoding putative GRs. The sequence sizes of *Agla*GR1, *Agla*GR5, and *Agla*GR10 ORFs were longer than 1,000 bp, indicating that they were nearly full-length genes. The other transcripts were too short to encode GRs. All but one locus (*AglaGR1* with a blast match with *Culex quinquefasciatus* [GenBank: XP_001848689.1]) had the best blast hit with *T. castaneum* (see supplementary Table S5).We observed four GR function lineages in the phylogenetic tree (Fig. 6). *Agla*GR1, *TcasTcas*GR178, *Tcas*GR1, *Tcas*GR2, *Tcas*GR3, *Dpon*GR1, *Dpon*GR3, *Ityp*GR3, *DmelDmel*GR21a and *Dmel*GR63a formed the first CO_2_ lineage. The second was the sugar lineage, which contained *Dmel*GR61a, *Dmel*GR64a, *Dmel*GR5a, *Dmel*GR64f, *Tcas*GR7, and *Amel*GR64f. *Dmel*GR33a, *Dmel*GR10a, and *Bmor*GR60 formed the third bitter taste receptor lineage. The last lineage was the fructose lineage, which contains *Dmel*GR43a, *Bmor*GR9, *Bmor*GR10, *Amel*GR43a, *Tcas*GR24, *Tcas*GR26, and *Tcas*GR27. Additionally, there were four Coleoptera specific lineages (red circles) and three Diptera lineages (blue circles) in the phylogenetic tree. The amino acid sequence identity among *Agla*PDEs ranged from 2.1% to 23.7%, with an average of 11.02%.

#### Fluorescence quantitative real-time PCR

**Figure 7:**
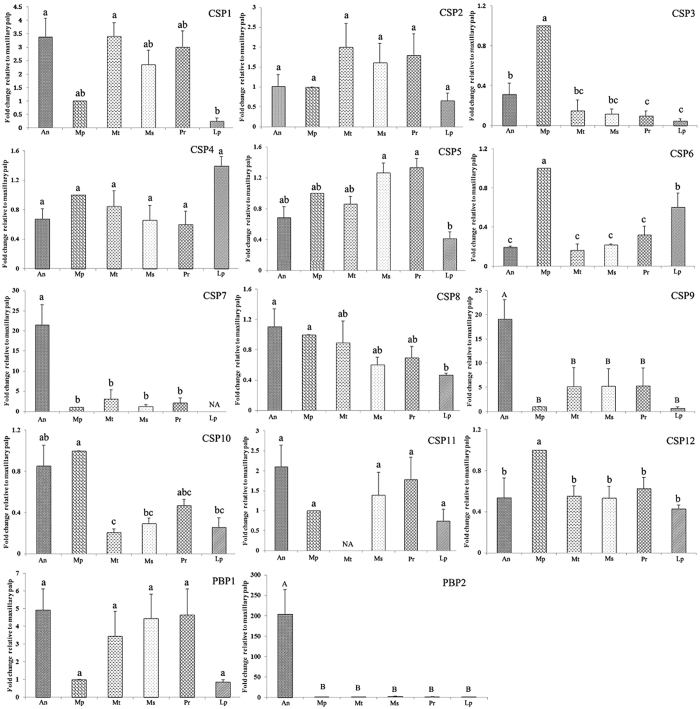
Chemosensory protein (CSPs) and pheromone-binding protein (PBPs) transcript levels in six tissues of *A. glabripennis.* An: antennae; Mp: maxillary palp; Mt: metapodium; Ms: mesopodium; Pr: propodium; Lp: labipalp. NA means the transcript level was too low to measure. β-Actin was used as the reference gene to normalize target gene expression. The standard errors are represented by error bars; different lowercase letters (a, b, c) above the bars denote significant differences at p < 0.05, and different capital letters (A, B) above bars denote significant differences at p < 0.01.

To verify the expression olfactory gene in antenna and characterize the expression profiles of chemosensory genes in six chemosensory tissues (antennae, propodiums, mesopodiums, metapedes, labipalps, and maxillary palps), 12 CSPs and 2 PBPs were selected for fluorescence quantitative real-time PCR. For both PBPs, we observed the highest expression level in antennae. Remarkably, *AglaPBP2* was expressed at a 200-fold higher level in antennae than in other tissues, and these differences were statistically highly significant. However, *AglaPBP1* expression in six tissues was not significantly different (Fig. 7). In CSPs, we observed the highest expression of *AglaCSP7*, *AglaCSP8*, *AglaCSP9*, and *AglaCSP11* in antennae. *The AglaCSP7* expression level was significantly higher in antennae than in all other tissues. Moreover, *AglaCSP9* expression level in antennae was highly significantly different from those in other tissue types. For *AglaCSP8* and *AglaCSP11*, the relative expression levels did not differ significantly between antennae and other tissues. The highest expression of *AglaCSP3, AglaCSP6*, *AglaCSP10* and *AglaCSP12* was observed in the maxillary palp. Remarkably, *AglaCSP3, AglaCSP6 and AglaCSP12* expression levels were significantly higher in antennae than in all other tissues. For labipalps, only *AglaCSP4* expression was highest, but the difference in the expression level among tissues was not significant (Fig. 7). High expression of *AglaCSP1*, *AglaCSP2*, *AglaCSP5*, *AglaCSP12* and *AglaPBP1* was detected in the foot (Fig. 7). Additionally, *AglaCSP1*, *AglaCSP2*, *AglaCSP5*, *AglaCSP8*, *AglaCSP10*, *AglaCSP12*, and *AglaPBP1* were expressed in multiple tissues; *AglaCSP1* was highly expressed in antennae and metapedes; *AglaCSP2* was highly expressed in propodiums, metapedes, and mesopodiums; *AglaCSP5* was highly expressed in propodiums and mesopodiums; *AglaCSP8* was highly expressed in all six tissues; *AglaCSP10* was highly expressed in antenna and maxillary palps; and *AglaCSP12* was highly expressed in all six tissues equally. Based on mean Cq values for CSPs and PBPs and the reference genes in six tissues, *AglaCSP1, AglaCSP2, AglaCSP5*, and *AglaCSP6* were highly expressed (Cq: 19–25, refs 22, 23, 24), *AglaCSP3, AglaCSP8, AglaCSP9*, and *AglaCSP12* exhibited intermediate expression (Cq: 25–31, refs 22, 23, 24), and *AglaCSP4, AglaCSP7, AglaCSP10, AglaCSP11*, and *AglaPBP1* exhibited low expression (Cq: 32–40, refs 22, 23, 24, 25, 26). The Cq of *AglaPBP2* ranged widely from 25 to 37.

## Discussion

Olfactory proteins have only been studied in 12 species in Coleoptera to date. The antenna transcriptome of ALB contributes provides important data for the identification of olfactory proteins in species in Coleoptera, and especially in species belonging to Cerambycidae, for which olfactory genes have only been identified in *M. alternatus*^39^,^40^ and *B. horsfieldi*^41^. Owing to the limited Coleoptera database, we observed several unigenes with best blast matches to a single accession number of the same species. In the ALB antenna transcriptome, about half of the olfactory genes had high sequence similarities to loci in *T. castaneum*, which has a well-studied genome with many genes encoding olfactory proteins^42^. Additionally, we were unable to categorize olfactory proteins into types, for instance, GOBP, ASP, and minus-C OBPs. Therefore, extensive research on olfactory genes is needed for species in Coleoptera, the largest insect order. In the ALB antenna transcriptome, we identified 42 OBPs, 12 CSPs, 14 PDEs, 37 ORs, 11 GRs, 2 SNMPs, and 4 IRs; this analysis substantially extends our knowledge of olfactory-related genes in coleopteran insects. Moreover, we confirmed the expression of all CSPs and PBPs in antennae, demonstrating that all chemosensory proteins are expressed in antennae and the authenticity of the ALB transcriptome. In addition, olfactory proteins participate in olfactory recognition that results in tree damage by stem-borers; accordingly, these results provide the basis for the development of novel strategies to manage one of the most damaging invasive forest borers.

OBPs are involved in the first critical step in odorant detection and are thought to interact with odorants in the sensillum lymph^10^,^43^. The 42 OBPs that we identified in the ALB antenna transcriptome was fewer than the 49 OBPs reported in the *T. castaneum* genome^42^ and the 52 transcripts encoding putative OBPs in the antennal transcriptomes of *M. alternatus* and its parasitoid *D. helophoroides*^39^. In the phylogenetic tree of OBPs, we observed two distant PBP lineages that were Coleoptera-specific and Lepidoptera-specific, which is consistent with the cladograms of all insect OBPs^10^. We speculate that PBPs in Coleoptera and Lepidoptera are conserved; however, among Coleoptera and Lepidoptera, PBPs showed variation. We detected antennae-biased expression of *AglaPBP2*, suggesting that antennae are the main pheromone recognition site, consistent with results of PBPs from *Sesamia nonagrioides* and *Helicoverpa assulta*; additionally, we found that PBP1–2 of *S. nonagrioides* and PBP1–3 of *H. assulta* are expressed at higher levels in male antennae than in female antennae; PBP3 of *S. nonagrioides* was expressed almost equally in both sexes^44^,^45^. *AglaPBP2* expression was 200-fold higher in antennae than in other tissues, indicating that it is specifically expressed in antennae. Moreover, a comparison of the Cq values of reference genes showed that *AglaPBP1* expression was low, and that *AglaPBP2* expression was high in antennae but low in other tissues, further suggesting that *AglaPBP2* is the main PBP involved in efficient and sensitive pheromone recognition. Analogously, tissue expression profiles of *Spodoptera litura* revealed that half of *SlitOBP* transcripts were expressed preferentially in antennae of both sexes, suggesting that they have a role in chemoreception^46^.

We identified 12 CSPs in the ALB transcriptome; this was fewer than the 16 CSP-encoding genes reported in *H. parallela*, 19 transcripts encoding putative CSPs in *M. alternatus* and *D. helophoroides*, and 20 CSPs reported in the *T. castaneum* genome. Of note, the phylogenetic tree of CSPs included one special clade that contained all CSPs of Diptera and was the order-specific CSP clade. The diversification of CSPs with insect order divergence was also observed in *Mamestra brassicae*^47^. Other than CSP11 in metapedes and CSP7 in labipalps, CSPs were expressed in all chemosensory tissues, reflecting their importance for insect sensory behavior. We observed clear antenna-biased CSP expression; two CSPs were expressed at significantly higher levels in antennae than in the other five tissue types, the same as the two most abundant CSPs in antennae of *Bombyx mori*^48^. In *Adelphocoris lineolatus* (Goeze), AlinCSP1–3 are mainly expressed in antennae^49^ and GmmCSP2 is transcribed at a very high level in the antennae of *Glossina morsitans*^50^. The maxillary palp-biased CSPs exhibited remarkable differences in expression compared with other tissues, consistent with results for *Vanessa gonerilla*^51^. In addition, sex-biased expression of some CSPs was detected; for instance, *GmmCSP2* was expressed at lower levels in male antennae than in female antennae^50^, and different sex-specific patterns of CSP1 and CSP2 expression have been observed in *B. mori*^48^. In addition to the six olfactory tissues, CSPs are also expressed in female pheromone glands, e.g., 7 CSPs in *B. mori*, and this might reflect their role in the solubilization of pheromone components and their delivery to the environment^48^. In addition, CSP expression in different growth phases has also been investigated. *GmmCSP2* exhibits a sharp increase in expression in 10-week-old flies^50^, and AlinCSP1–3 are highly expressed in 5^th^-instar nymphs^49^.

The phylogenetic tree of PDE was not based on order (i.e., Coleoptera or Lepidoptera), but on ODEs and PDEs (except for SinODE-CEX19). The phylogenetic tree of SNMPs indicated the differentiation process of them was function differentiation, not according with species differentiation. Additionally, we identified more PDEs than the two PBPs and three ALB pheromone compounds. We speculate that each PDE may act with one specific, single odorant or odorant-OR/PR complex. Compared with PDEs, ODEs have more combinations of odorant-OR/PR complexes. Three SNMPs have been identified in insect species, such as in *I. typographus* and *D. ponderosae*. However, we identified two SNMPs in ALB, which is similar to the number in *T. molitor*. In the phylogenetic tree, all SNMPs from Coleoptera, Diptera, Lepidoptera and Hymenoptera clustered into two clades, SNMP1 and SNMP2, which are the same as the two SNMP clades identified by phylogenetic analysis in *I. typographus* and *D.ponderosae*^52^.

We identified 37 transcripts encoding ORs in the ALB transcriptome, which is fewer than the 43 known ORs in *I. typographus*, 49 ORs in *D. ponderosae,* 57 ORs in *M. caryae*, 43 ORs in *A. corpulenta,* and 111 ORs in *T. castaneum*. In the phylogenetic tree of ORs, the specific Orco lineage contained *Agla*OR9, which shows that *Agla*OR9 has high similarity with five Coleoptera Orco and *Dmel*Orco, and that *Agla*OR9 could be the Orco of *A.* g*labripennis*. Additionally, most ORs were clustered into more than ten lineages among the orders, but the evolutionary relationships between them were complicated. Four ionotropic receptors were identified. In the phylogenetic tree of IRs, IR25a and IR8a formed a conserved IR clade, in agreement with the results of *Helicoverpa armigera* and *H.assulta*^45^. *AglaIR4* was in the conserved IR clade, which indicates that *AglaIR4* is the conserved IR of *A.* g*labripennis*. The number of ionotropic receptors of *A.* g*labripennis* (4) was fewer than in almost all Coleoptera species (15 in *D. ponderosae*, 9 oin *Colaphellus bowringi*, 8 in *Rhyzopertha dominica*, 7 in *I. typographus*, 6 in *Tenebrio molitor*, 5 in *Anomala corpulenta,* and 3 in *Dendroctonus valens*) (NCBI: http://www.ncbi.nlm.nih.gov/), which may due to the lower expression of IRs in *A.* g*labripennis* than in other Coleoptera species^52^. No ceoloconic sensilla are present in *A.* g*labripennis* antenna^53^.

According to the search results in NCBI (http://www.ncbi.nlm.nih.gov/), the number of IRs in Lepidoptera species (generally more than 9) and Diptera species (generally more than 11) is more than in Coleoptera species (generally less than 9), and is especially lower than in Lepidoptera species and *D. melanogaster* (148), which may because ceoloconic sensilla have more locations in Lepidoptera^54^ and Diptera species^55^. Eleven transcripts encoding putative GRs were identified. As CO2, sugar, fructose and bitter receptors formed the GR phylogenetic tree, this indicated that *Agla*GR1 may detect CO2 in *A. glabripennis*. The sugar and fructose receptors lineages were consistent with the functions of *Bmor*GR8 and *Bmor*GR9^56^,^57^.

## Methods

### Insect and tissue collection

ALB (*A. glabripennis* Motsch) larvae were collected from damaged *Populus* species that were chopped down and brought to the laboratory at the end of May 2013 in Beijing, China. The insects were fed poplar short-cut wood from their natural environment. Antennae from males and females were excised and stored in RNAlater (Ambion, Austin, TX, USA) at −80 °C. All operations were performed according to ethical guidelines to minimize pain and discomfort to the insects.

### cDNA library construction and Illumina sequencing

Total RNA was extracted from two female and two male antennae using TRIzol reagent (Ambion) and the RNeasy Plus Mini Kit (No. 74134; Qiagen, Hilden, Germany) following the manufacturer’s instructions. RNA quantity was detected using the NanoDrop 8000 (Thermo, Waltham, MA, USA). A quarter of the total RNA for the antennae of each male and female was mixed and used to construct the cDNA library. The cDNA library construction and Illumina sequencing of samples were performed at CapitalBio Corporation (Beijing, China). The mRNA samples were purified and fragmented using the TruSeq RNA Sample Preparation Kit v2-Set A (No. RS-122-2001; Illumina, San Diego, CA, USA). Random hexamer primers were used to synthesize the first-strand cDNA, followed by synthesis of the second-strand cDNA using buffer, dNTPs, RNase H, and DNA polymerase I at 16 °C for 1 h. After end repair, A-tailing, and the ligation of adaptors, the products were amplified by PCR and quantified precisely using the Qubit DNA Br Assay Kit (Q10211; Invitrogen, Carlsbad, CA, USA). They were then purified using the MinElute Gel Extraction Kit (Qiagen, Cat No. 28604) to obtain a cDNA library. The cDNA library was sequenced on the HiSeq2000 platform.

### Assembly and functional annotation

*De novo* transcriptome assembly was carried out with the short-read assembly program Trinity (Version: r2013-11-10). All raw reads were processed to remove low-quality and adaptor sequences. The clean reads were assembled using the default parameters. The largest alternative splicing variants in the Trinity results were called unigenes. The annotation of unigenes was performed by NCBI BLASTx searches against the Nr protein database, with an E-value threshold of 1e-5. The blast results were then imported into the Blast2GO pipeline for GO annotation. The longest ORF for each unigene was determined by the NCBI ORF Finder tool (http://www.ncbi.nlm.nih.gov/gorf/gorf.html).

### Identification of chemosensory genes

With tBLASTn, the available sequences of OBP, CSP, OR, GR, IR, and SNMP proteins from Insecta species were used as queries to identify candidate unigenes involved in olfaction in *A. glabripennis*. All candidate OBPs, CSPs, ORs, GRs, IRs, and SNMPs were manually checked by assessing the NCBI BLASTx results.

### Sequence and phylogenetic analysis

The candidate OBPs and PBPs were searched for the presence of N-terminal signal peptides using SignalP4.0 (http://www.cbs.dtu.dk/services/SignalP/) and the transmembrane domains of candidate ORs, IRs, GRs, and SNMPs were predicted using the TMHMM server v3.0 (http://www.cbs.dtu.dk/services/TMHMM/). Amino acid sequence alignment was performed using the ClustalW method implemented in the Mega v6.0 software package^58^. The phylogenetic tree was constructed using the neighbor-joining (NJ) method^59^ with P-distances model and pairwise deletion of gaps was performed in Mega v6.0 software package. The reliability of the tree structure and node support was evaluated by bootstrap analysis with 1000 replicates. To obtain a phylogenetic tree with higher bootstrap support, sequences of binding proteins (full length gene 400 bp; OBPs and CSPs) less than 390 bp and redundant sequences were removed; sequences of receptor proteins (full length genes at least more than 1000 bp; ORs, IRs and GRs) with genome less than 1200 bp and others less than 900 bp and redundant sequences were removed. The phylogenetic trees were colored and rearranged in FigTree v1.4.2. The phylogenetic analyses of *Agla*OBPs, *Agla*CSPs, *Agla*ORs, *Agla*IRs, *Agla*GRs, *Agla*SNMPs, and *Agla*PDEs were based on the amino sequences of the putative chemosensory genes and the sequences of homologous genes in *Drosophila melanogaster, Bombyx mori, Apis mellifera,* and other species of Coleoptera. The protein names and gene accession numbers are provided in supplementary Table S7.

### Expression analysis by fluorescence quantitative real-time PCR

Fluorescence quantitative real-time PCR was performed to verify the expression of candidate chemosensory genes. Antennae, foot (including the propodium, mesopodium, and metapedes), labipalp, and maxillary palps were collected from two male and two female adult ALB for each biological replicate. Total RNA was extracted following the methods described above and was used as template for cDNA synthesis using the PrimeScript RT Reagent Kit with gDNA Eraser (No. RR047A; TaKaRa, Shiga, Japan). RNA from each tissue used to synthesize cDNA was a mixture of equal amounts of RNA from four ALBs. Gene-specific primers were designed using Primer3 (http://bioinfo.ut.ee/primer3-0.4.0/) (see supplementary Table S6). Actin was identified from the ALB antennal transcriptome and used as a reference gene. A PCR analysis was conducted using the Bio-Rad CFX96 PCR System (Hercules, CA, USA). SYBR Premix Ex Taq™ II (No. RR820A; TaKaRa) was used for the PCR reaction using three-step amplification. Each PCR reaction was conducted in a 25-ml reaction mixture containing 12.5 μl of SYBR Premix Ex Taq II, 1 ml of each primer (10 mM), 2 μl of sample cDNA (2.5 ng of RNA), and 8.5 μl of dH2O (sterile distilled water). The RT-qPCR cycling parameters were as follows: 95 °C for 30 s, followed by 40 cycles of 95 °C for 5 s, 60 °C for 30 s, and 65 °C to 95 °C in increments of 0.5 °C for 5 s to generate the melting curves. To examine reproducibility, each qPCR reaction for each tissue was performed in three technical replicates and three biological replicates. Negative controls without either template were included in each experiment. Bio-Rad CFX Manager (version 3.1.1517.0823) was used to normalize expression based on ΔΔCq values, with maxillary palps in analyze mode as control samples, and the 2^−ΔΔCT^ method was used (the amplification efficiency for 14 genes was equal to 100%)^60^. The comparative analyses for every gene among six tissue types were assessed by a one-way nested analysis of variance (ANOVA), followed by Tukey’s honestly significance difference (HSD) tests implemented in SPSS Statistics 18.0. Values are presented as means ± SE.

## Additional Information

**How to cite this article**: Hu, P. *et al.* Antennal transcriptome analysis of the Asian longhorned beetle *Anoplophora glabripennis*. *Sci. Rep.* **6**, 26652; doi: 10.1038/srep26652 (2016).

## Supplementary Material

Supplementary Information

## Figures and Tables

**Table 1:** Best blastx hits for putative chemosensory proteins of *Anoplophora glabripennis.*

Number	Gene ID	Unigene Length (bp)	ORF Length (bp)	Complete ORF	Signal Peptide	Cysteine Number	FPKM	Best Blastx Match					
								Name	Acc. number	Species	Score	E-value	Identity (%)
CSP1	Unigene16278	1291	462	Y	Y	5	11190.0	chemosensory protein	AEC04843.1	*Batocera horsfieldi*	169	4.00E-47	92%
CSP2	Unigene6655	742	390	Y	Y	5	2873.9	chemosensory protein	AEC04842.1	*Batocera horsfieldi*	176	5.00E-52	87%
CSP3	Unigene5172	490	384	Y	Y	5	61.8	chemosensory protein 3	AIT38536.1	*Bemisia tabaci*	147	9.00E-42	62%
CSP4	Unigene5579	677	504	Y	N	9	51.0	chemosensory protein 2	AGI05172.1	*Dendroctonus ponderosae*	141	1.00E-38	50%
CSP5	Unigene5322	529	414	Y	Y	4	614.7	chemosensory protein 1	AGI05161.1	*Dendroctonus ponderosae*	144	1.00E-40	53%
CSP6	Unigene5135	534	381	Y	Y	4	1498.4	chemosensory protein 8	AGI05164.1	*Dendroctonus ponderosae*	158	6.00E-46	60%
CSP7	Unigene11951	802	417	Y	Y	4	281.8	chemosensory protein	AFI45003.1	*Dendroctonus ponderosae*	207	9.00E-64	68%
CSP8	Unigene14677	2686	933	Y	Y	5	29.9	chemosensory protein 6 precursor	NP_001039288.1	*Tribolium castaneum*	245	4.00E-49	54%
CSP9	Unigene9546	619	381	Y	Y	4	126	chemosensory protein 12 precursor	NP_001039280.1	*Tribolium castaneum*	150	3.00E-42	59%
CSP10	Unigene4446	852	372	Y	Y	5	2.5	chemosensory protein 7 precursor	NP_001039289.1	*Tribolium castaneum*	200	8.00E-61	77%
CSP11	Unigene12790	1052	303	Y	Y	4	11.1	chemosensory protein 8 precursor	NP_001039290.1	*Tribolium castaneum*	137	4.00E-36	78%
CSP12	Unigene10121	854	450	Y	Y	5	132.3	chemosensory protein 5 precursor	NP_001039287.1	*Tribolium castaneum*	149	1.00E-40	56%
