# Supplementary material for: Antennal transcriptome analysis of the Asian longhorned beetle Anoplophora glabripennis
Source: Sci Rep. 2016 May 25;6:26652. doi: 10.1038/srep26652 (PMC4879564; doi:10.1038/srep26652)
Supplement: Supplementary Information [file srep26652-s1.pdf]

Antennal transcriptome analysis of the Asian longhorned beetle *Anoplophora glabripennis*

Ping Hu, Jingzhen Wang , Mingming Cui, Jing Tao*, Youqing Luo*

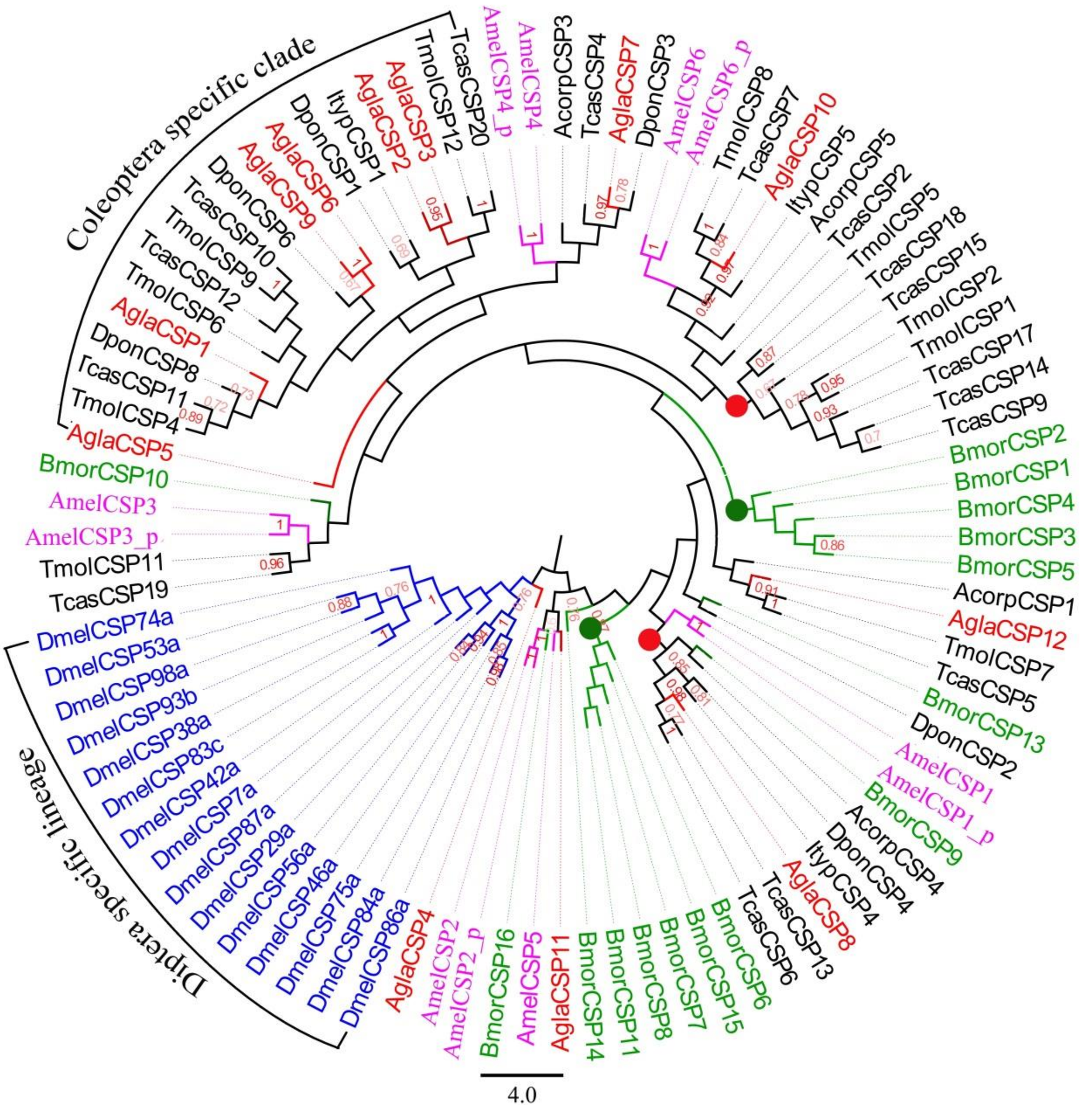

Supplementary FigureS1 Neighbor-joining phylogenetic tree of candidate chemosensory proteins (CSPs)

The NJ phylogenetic analysis of CSPs of *A. glabripennis* (*Agla*CSP, red) was performed with reference CSPs of *Tenebrio molitor* (*Tmol*CSP, dark), *D. ponderosae* (*Dpon*CSP, dark), *I. typographus* (*Ityp*CSP, dark), *T. castaneum* (*Tcas*CSP, dark), *Drosophila melanogaster* (*Dmel*CSP, Diptera, blue), *Bombyx mori* (*Bmor*CSP, Lepidoptera, green), *Apis mellifera* (*Amel*CSP, Hymenoptera, purple). The stability of the nodes was assessed by bootstrap analysis with 1,000 replications, and only bootstrap values ≥0.6 are shown at the corresponding nodes. The scale bar represents 4.0 substitutions per site.

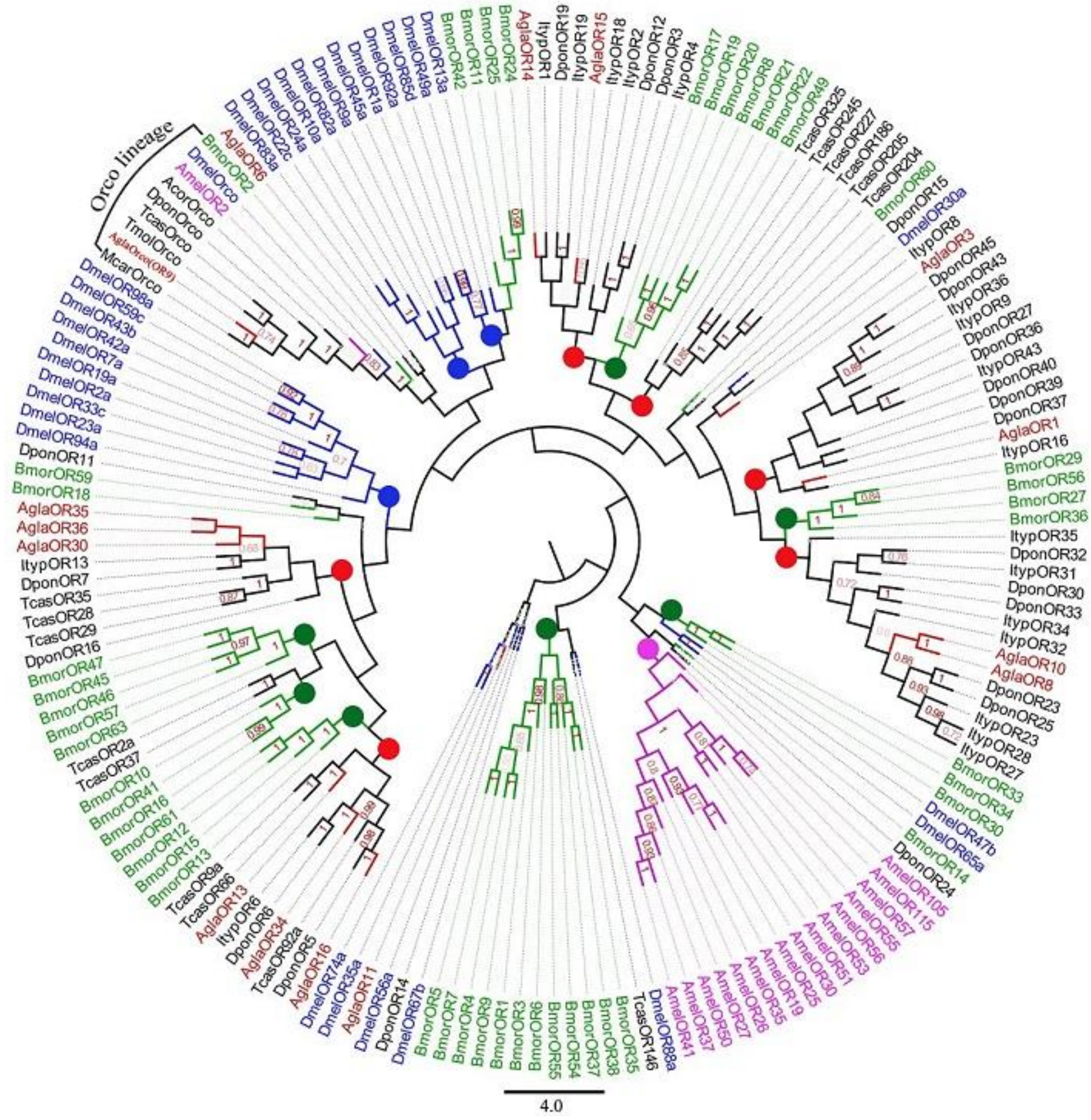

Supplementary Figure S2 Neighbor-joining phylogenetic tree of candidate odorant receptors (ORs)

The NJ phylogenetic analysis of ORs of *A. glabripennis* (*Agla*OR, red) was performed with reference ORs of *D. ponderosae* (*Dpon*OR, dark), *I. typographus* (*Ityp*OR, dark), *T. castaneum* (*Tcas*OR, dark), *Tenebrio molitor* (*TmolOrco*, dark)， *Anomala corpulenta*(*AcorOrco*, dark)， *Megacyllene caryae*(*McarOrco*, dark), *D. melanogaster* (*Dmel*OR, Diptera, blue), *B. mori* (*Bmor*OR, Lepidoptera, green), *A. mellifera* (*Amel*OR, Hymenoptera, purple) The stability of the nodes was assessed by bootstrap analysis with 1,000 replications, and only bootstrap values ≥0.6 are shown at the corresponding nodes. The scale bar represents 4.0 substitution per site.

Supplementary Table S1 Best blastx hits for putative odorant-binding proteins of *Anoplophora glabripennis*

								Best Blastx Match					
**Number**	**Gene ID**	**Unigene Length (bp)**	**ORF Length (bp)**	**Complete ORF**	**Signal Peptide**	**Cysteine Number**	**FPKM**	**Name**	**Acc. number**	**Species**	**Score**	**E-value**	**Identity (%)**
OBP1	Unigene11459	586	477	Y	Y	6	510.8	minus-C odorant binding protein 3	ADD82416.1	*Batocera horsfieldi*	79.3	3.00E-15	32%
OBP2	Unigene16298	717	414	Y	Y	7	1055.8	odorant-binding protein 2	AHA33380.1	*Batocera horsfieldi*	239	1.00E-76	81%
OBP3	Unigene6991	509	381	Y	Y	6	372.7	odorant-binding protein 3	AHA33381.1	*Batocera horsfieldi*	97.1	2.00E-22	46%
OBP4	Unigene6891	305	303	N	Y	4	277.1	minus-C odorant binding protein 3	ADD82416.1	*Batocera horsfieldi*	55.8	1.00E-07	34%
OBP5	Unigene10203	620	405	Y	Y	7	228.4	minus-C odorant binding protein 3	ADD82416.1	*Batocera horsfieldi*	153	2.00E-43	64%
OBP6	Unigene17306	479	408	Y	Y	4	1.4	minus-C odorant binding protein 4	ADD82417.1	*Batocera horsfieldi*	118	2.00E-30	48%
OBP7	Unigene18489	410	405	N	Y	4	0.5	minus-C odorant binding protein 4	ADD82417.1	*Batocera horsfieldi*	118	2.00E-30	48%
OBP8	Unigene8141	534	387	Y	Y	6	47.6	minus-C odorant binding protein 4	ADD82417.1	*Batocera horsfieldi*	73.9	2.00E-13	36%
OBP9	Unigene20262	370	357	N	Y	5	0.9	minus-C odorant binding protein 4	ADD82417.1	*Batocera horsfieldi*	71.2	5.00E-13	41%
OBP10	Unigene6534	697	429	Y	Y	7	967.7	minus-C odorant binding protein 4	ADD82417.1	*Batocera horsfieldi*	81.6	6.00E-16	36%
OBP11	Unigene9135	447	428	N	N	6	6.3	minus-C odorant binding protein 4	ADD82417.1	*Batocera horsfieldi*	71.2	8.00E-13	38%
OBP12	Unigene11630	586	423	Y	Y	5	174.7	minus-C odorant binding protein 3	ADD82416.1	*Batocera horsfieldi*	199	1.00E-61	68%
OBP13	Unigene7905	740	411	Y	Y	5	1532.1	minus-C odorant binding protein 3	ADD82416.1	*Batocera horsfieldi*	241	3.00E-77	88%
OBP14	Unigene9526	767	447	Y	Y	4	466.0	minus-C odorant binding protein 2	ADD70031.1	*Batocera horsfieldi*	131	4.00E-34	42%
OBP15	Unigene10687	565	435	Y	N	5	4136.2	minus-C odorant binding protein 2	ADD70031.1	*Batocera horsfieldi*	155	3.00E-44	53%
OBP16	Unigene8545	480	435	Y	Y	4	11.8	minus-C odorant binding protein 2	ADD70031.1	*Batocera horsfieldi*	144	2.00E-40	50%
OBP17	Unigene11277	598	435	Y	Y	4	16103.1	minus-C odorant binding protein 2	ADD70031.1	*Batocera horsfieldi*	150	3.00E-42	56%
OBP18	Unigene9512	584	390	Y	Y	4	1103.7	minus-C odorant binding protein 1	ADD70030.1	*Batocera horsfieldi*	150	2.00E-42	55%
OBP19	Unigene9293	607	408	Y	N	7	7.7	pheromone binding protein PBP2	AIV43009.1	*Batocera horsfieldi*	109	1.00E-26	49%
OBP20	Unigene10710	822	411	Y	N	9	100.6	pheromone binding protein PBP1	AIV43008.1	*Batocera horsfieldi*	200	1.00E-60	74%

OBP21	Unigene9373	905	486	Y	N	7	126.2	odorant-binding protein 29	AGI05182.1	*Dendroctonus ponderosae*	105	5.00E-24	43%
OBP22	Unigene9457	867	645	Y	Y	9	135.3	odorant-binding protein 20	AGI05168.1	*Dendroctonus ponderosae*	149	9.00E-40	37%
OBP23	Unigene13779	904	738	Y	Y	12	49.9	odorant-binding protein 2	AGI05158.1	*Dendroctonus ponderosae*	163	2.00E-44	40%
OBP24	Unigene4719	384	351	Y	Y	8	9.0	odorant-binding protein 16	AGI05186.1	*Dendroctonus ponderosae*	57.8	3.00E-08	31%
OBP25	Unigene11363	597	432	Y	Y	9	97.3	odorant-binding protein	AFI45063.1	*Dendroctonus ponderosae*	155	3.00E-44	50%
OBP26	Unigene13800	1237	501	Y	Y	8	59.0	odorant binding protein	AII00979.1	*Dendrolimus houi*	90.5	3.00E-18	46%
OBP27	Unigene4787	416	258	Y	N	3	4.1	odorant-binding protein 2	AHA39267.1	*Monochamus alternatus*	114	2.00E-29	42%
OBP28	Unigene9544	1321	405	Y	Y	5	2257.7	odorant-binding protein 2	AHA39267.1	*Monochamus alternatus*	221	6.00E-67	91%
OBP29	Unigene9517	613	423	Y	N	8	1078.6	odorant-binding protein 3	AHA39268.1	*Monochamus alternatus*	247	4.00E-80	84%
OBP30	Unigene6697	437	414	Y	N	4	1.5	odorant binding protein 1	ABR53888.1	*Monochamus alternatus*	108	7.00E-27	43%
OBP31	Unigene15661	1928	186	Y	N	7	80.6	odorant binding protein 1	ABR53888.1	*Monochamus alternatus*	105	7.00E-23	55%
OBP32	Unigene6515	752	429	Y	Y	9	696.0	odorant-binding protein 4	AHA39269.1	*Monochamus alternatus*	256	5.00E-83	85%
OBP33	Unigene4720	524	423	Y	Y	5	10.5	odorant-binding protein 2	AHA39267.1	*Monochamus alternatus*	85.9	6.00E-18	40%
OBP34	Unigene13204	465	405	Y	Y	5	9.6	odorant-binding protein 2	AHA39267.1	*Monochamus alternatus*	98.6	6.00E-23	48%
OBP35	Unigene6308	343	117	N	N	1	6.8	odorant binding protein 6	AJO67868.1	*Monochamus alternatus*	43.5	3.00E-03	56%
OBP36	Unigene5103	1279	537	Y	Y	9	158.7	odorant binding protein 9	AGZ04909.1	*Sogatella furcifera*	174	3.00E-48	53%
OBP37	Unigene6425	440	432	N	N	7	1.1	orant-binding protein 9	AJM71483.1	*Tenebrio molitor*	214	6.00E-68	78%
OBP38	Unigene25353	335	324	N	N	6	0.4	odorant binding protein C20	EFA01425.1	*Tribolium castaneum*	87.8	2.00E-19	41%
OBP39	Unigene10918	827	429	Y	Y	6	10.9	odorant binding protein C20	EFA01425.1	*Tribolium castaneum*	96.3	6.00E-21	50%
OBP40	Unigene4551	625	429	Y	Y	4	3.4	odorant binding protein C03	EFA07546.1	*Tribolium castaneum*	62	4.00E-09	31%
OBP41	Unigene11194	493	405	Y	Y	10	13.4	odorant binding protein 19	AJM71493.1	*Tenebrio molitor*	152	2.00E-43	51%
OBP42	Unigene12097	662	525	Y	N	5	3435.9	odorant binding proteinC01	EFA07544.1	*Tribolium castaneum*	72.4	6.00E-16	34%

Supplementary TableS2 Best blastx hits for putative pheromone-degrading enzymes of *Anoplophora glabripennis*

						Best Blastx Match					
**Number**	**Gene ID**	**Unigene Length(bp)**	**ORF Length(bp)**	**Complete ORF**	**FPKM**	**Name**	**Acc. number**	**Species**	**Score**	**E-value**	**Identity (%)**
PDE1	Unigene11785	1630	1470	Y	40.1	pheromone-degrading enzyme	AAT38512.1	*Phyllopertha diversa*	338	1.00E-105	41%
PDE2	Unigene13379	1159	528	Y	34.1	pheromone-degrading enzyme	AAT38512.1	*Phyllopertha diversa*	234	6.00E-68	43%
PDE3	Unigene14171	2061	336	Y	101.1	pheromone-degrading enzyme	AAT38512.1	*Phyllopertha diversa*	136	1.00E-30	51%
PDE4	Unigene16871	619	488	N	2.4	pheromone-degrading enzyme	AAT38512.1	*Phyllopertha diversa*	163	8.00E-44	86%
PDE5	Unigene5660	472	444	N	10.0	pheromone-degrading enzyme	AAT38512.1	*Phyllopertha diversa*	121	2.00E-29	42%
PDE6	Unigene9926	660	428	Y	26.3	pheromone-degrading enzyme	AAT38512.1	*Phyllopertha diversa*	183	4.00E-52	61%
PDE7	Unigene11466	1860	1695	Y	11.0	pheromone-degrading enzyme	AAX58713.1	*Popillia japonica*	453	1.00E-148	42%
PDE8	Unigene11140	468	394	N	14.5	pheromone-degrading enzyme	AAX58713.2	*Popillia japonica*	84	8.00E-16	38%
PDE9	Unigene11275	435	431	N	9.4	pheromone-degrading enzyme	AAX58713.3	*Popillia japonica*	133	1.00E-33	46%
PDE10	Unigene12385	1829	1689	Y	201.5	pheromone-degrading enzyme	AAX58713.4	*Popillia japonica*	372	2.00E-117	39%
PDE11	Unigene12911	987	971	N	3.9	pheromone-degrading enzyme	AAX58713.5	*Popillia japonica*	269	1.00E-81	45%
PDE12	Unigene13151	2134	1668	Y	3.0	pheromone-degrading enzyme	AAX58713.6	*Popillia japonica*	479	2.00E-157	44%
PDE13	Unigene14073	1198	1140	Y	41.9	pheromone-degrading enzyme	AAX58713.7	*Popillia japonica*	307	3.00E-95	43%
PDE14	Unigene14607	1224	1182	N	11.3	pheromone-degrading enzyme	AAX58713.8	*Popillia japonica*	386	2.00E-125	48%
ODE1	Unigene12777	551	339	Y	4.1	odorant degrading enzyme CXE13	AII21987.1	*Sesamia inferens*	82	7.00E-15	31%

Supplementary Table S3 Best blastx hits for putative odorant receptors of *Anoplophora glabripennis*

Number	Gene ID	Unigene Length (bp)	ORF Length (bp)	Complete ORF	TM Number	FPKM	Best Blastx Match					
							Name	Acc. number	Species	Score	E-value	Identity (%)
OR1	Unigene10234	1347	1158	Y	7	1.8	olfactory receptor, putative	XP_001651755.1	*Aedes aegypti*	72.4	1.00E-10	24%
OR2	Unigene17619	481	276	Y	1	1.6	odorant receptor	XP_001651754.1	*Aedes aegypti*	74.7	6.00E-13	43%
OR3	Unigene6256	1287	1197	Y	6	1.1	odorant receptor 2	ACH69148.1	*Anopheles stephensi*	88.2	7.00E-16	29%
OR4	Unigene19030	313	225	Y	0	0.8	TPA: olfactory receptor 48	DAA34890.1	*Bombyx mori*	53.9	3.00E-06	33%
OR5	Unigene22099	326	222	Y	1	0.7	olfactory receptor	BAH66342.1	*Bombyx mori*	53.5	5.00E-06	34%
OR6	Unigene17432	473	354	Y	0	1.6	odorant receptor	AID61215.1	*Calliphora stygia*	52.8	2.00E-05	30%
OR7	Unigene18206	510	345	Y	1	0.8	odorant receptor 23	AGI05173.1	*Dendroctonus ponderosae*	84.7	2.00E-16	35%
OR8	Unigene17758	1113	996	Y	4	0.9	odorant receptor 23	AGI05173.1	*Dendroctonus ponderosae*	187	2.00E-51	35%
Orco (OR9)	Unigene13568	2824	1434	Y	10	17.9	olfactory receptor	AFI45064.1	*Dendroctonus ponderosae*	806	0	84%
OR10	Unigene14393	1436	1209	Y	4	4.5	odorant receptor 23	AGI05173.1	*Dendroctonus ponderosae*	200	6.00E-55	31%
OR11	Unigene18618	1009	768	Y	4	0.8	odorant receptors	AII01107.1	*Dendrolimus kikuchii*	78.2	1.00E-12	23%
OR12	Unigene8715	406	372	Y	3	1.4	odorant receptors	AII01107.1	*Dendrolimus kikuchii*	53.9	6.00E-06	30%
OR13	Unigene9386	1212	1026	Y	6	1.5	olfactory receptor 15	CAM84013.1	*Tribolium castaneum*	139	8.00E-34	37%
OR14	Unigene9004	1331	1155	Y	6	2.2	odorant receptor 95	EFA10670.1	*Tribolium castaneum*	204	4.00E-57	28%
OR15	Unigene8797	1337	1158	Y	8	1.5	odorant receptor 89	EFA10702.1	*Tribolium castaneum*	182	5.00E-49	32%
OR16	Unigene7215	703	600	Y	2	3.5	odorant receptor 64	EFA10800.1	*Tribolium castaneum*	138	8.00E-35	35%
OR17	Unigene6163	681	459	Y	3	1.0	odorant receptor 47	EFA02940.1	*Tribolium castaneum*	108	3.00E-24	34%
OR18	Unigene6089	634	617	N	4	0.7	odorant receptor 123	EEZ99420.1	*Tribolium castaneum*	55.8	4.00E-06	26%
OR19	Unigene3103	660	570	Y	0	1.3	odorant receptor 61	EEZ99416.1	*Tribolium castaneum*	132	1.00E-32	35%
OR20	Unigene25662	324	303	N	3	0.4	odorant receptor 52	EFA02940.1	*Tribolium castaneum*	64.7	6.00E-10	37%

OR21	Unigene23567	375	357	N	2	0.5	odorant receptor 92	EFA02873.1	*Tribolium castaneum*	70.5	8.00E-12	34%
OR22	Unigene23356	302	216	Y	0	0.8	odorant receptor 167	EFA02801.1	*Tribolium castaneum*	84.3	5.00E-17	44%
OR23	Unigene22827	303	261	N	1	0.5	odorant receptor 114	EFA05790.1	*Tribolium castaneum*	52.8	7.00E-06	35%
OR24	Unigene22024	368	351	Y	0	0.6	odorant receptor 119	EEZ99418.1	*Tribolium castaneum*	55.8	9.00E-07	30%
OR25	Unigene20712	442	387	N	3	0.5	odorant receptor 128	EFA02867.1	*Tribolium castaneum*	52	3.00E-05	31%
OR26	Unigene20672	401	381	N	2	0.6	odorant receptor 100	EFA13313.1	*Tribolium castaneum*	73.9	8.00E-13	32%
OR27	Unigene20574	551	495	N	2	0.9	odorant receptor 123	EEZ99420.1	*Tribolium castaneum*	60.5	8.00E-08	28%
OR28	Unigene20290	460	444	N	2	0.8	odorant receptor 128	EFA02867.1	*Tribolium castaneum*	50.4	1.00E-04	27%
OR29	Unigene20049	473	384	Y	3	0.5	odorant receptor 64	EFA10800.1	*Tribolium castaneum*	144	3.00E-38	52%
OR30	Unigene19434	943	756	Y	4	0.9	odorant receptor 61	EEZ99416.1	*Tribolium castaneum*	254	3.00E-78	45%
OR31	Unigene18988	328	285	Y	2	1.3	odorant receptor 42	EEZ99226.1	*Tribolium castaneum*	85.9	2.00E-17	36%
OR32	Unigene17964	548	495	N	3	1.3	odorant receptor 167	EFA02801.1	*Tribolium castaneum*	93.2	3.00E-19	31%
OR33	Unigene17303	635	357	Y	0	1.2	odorant receptor 3	EFA01310.1	*Tribolium castaneum*	114	3.00E-26	40%
OR34	Unigene17240	1121	864	Y	6	1.2	odorant receptor 64	EFA10800.1	*Tribolium castaneum*	279	7.00E-87	48%
OR35	Unigene16603	1064	954	Y	4	2.6	odorant receptor 59	EEZ99416.1	*Tribolium castaneum*	201	4.00E-57	34%
OR36	Unigene15115	1432	1170	Y	7	3.6	odorant receptor 59	EEZ99416.1	*Tribolium castaneum*	313	1.00E-98	42%
OR37	Unigene1267	462	285	Y	3	0.8	odorant receptor 42	EEZ99226.1	*Tribolium castaneum*	96.7	7.00E-21	39%

Supplementary Table S4 Best blastx for ionotropic receptors of *Anoplophora glabripennis*

							Best Blastx Match					
**Number**	**Gene ID**	**Unigene Length (bp)**	**ORF Length (bp)**	**Complete ORF**	**TM Number**	**FPKM**	**Name**	**Acc.number**	**Species**	**Score**	**E-value**	**Identity (%)**
IR1	unigene6086	752	720	N	0.48	3	chemosensory ionotropic receptor 21a	AKC58586.1	*Anomala corpulenta*	313	2.00E-97	58%
IR2	unigene6102	1619	1398	Y	1.51	3	ionotropic receptor IR2	ALR72541.1	*Colaphellus bowringi*	704	0	69%
IR3	unigene4244	743	681	N	1.09	2	putative ionotropic receptor IR64a	ALR72584.1	*Colaphellus bowringi*	303	3.00E-100	77%
IR4	unigene16073	2761	2466	Y	12.94	3	chemosensory ionotropic receptor x	AKC58590.1	*Anomala corpulenta*	442	8.00E-137	34%

Supplementary Table S5 Best blastx for gustatory receptors and sensory neuron membrane proteins of *Anoplophora glabripennis*

							Best Blastx Match					
**Number**	**Gene ID**	**Unigene Length(bp)**	**ORF Length(bp)**	**Complete ORF**	**TM Number**	**FPKM**	**Name**	**Acc. number**	**Species**	**Score**	**E-value**	**Identity (%)**
GR1	Unigene12068	1454	1344	Y	7	3.2	gustatory receptor 24	XP_001848689.1	*Culex quinquefasciatus*	433	3.00E-144	50%
GR2	Unigene7839	668	435	Y	0	48.7	gustatory receptor candidate 25	CAL23158.2	*Tribolium castaneum*	113	4.00E-26	47%
GR3	Unigene26342	412	348	N	3	0.5	gustatory receptor candidate 29	CAL23162.2	*Tribolium castaneum*	93.6	8.00E-20	37%
GR4	Unigene26042	345	300	N	3	0.4	gustatory receptor candidate 40	CAL23173.2	*Tribolium castaneum*	84.7	4.00E-17	35%
GR5	Unigene18233	1073	1062	N	7	1.0	gustatory receptor candidate 58	CAL23191.2	*Tribolium castaneum*	60.1	1.00E-06	30%
GR6	Unigene19269	850	846	N	5	0.7	gustatory receptor candidate 58	CAL23191.2	*Tribolium castaneum*	60.5	3.00E-07	31%
GR7	Unigene7881	478	254	Y	0	0.5	gustatory receptor 102	EFA02935.1	*Tribolium castaneum*	63.9	4.00E-09	33%
GR8	Unigene20071	303	269	N	2	0.8	gustatory receptor 5	EFA04711.1	*Tribolium castaneum*	112	5.00E-27	58%
GR9	Unigene2799	311	273	N	1	0.6	gustatory receptor 24	EFA05759.1	*Tribolium castaneum*	72.8	1E-12	46%
GR10	Unigene14056	1389	1182	Y	7	12.6	gustatory receptor Gr83	NP_001138948.1	*Tribolium castaneum*	380	1.00E-124	54%
GR11	Unigene8396	461	321	Y	1	3.9	gustatory receptor Gr109	NP_001138957.1	*Tribolium castaneum*	51.6	3.00E-05	24%
SNMP1	Unigene16260	5911	1686	Y	2	44.02	sensory neuron membrane protein 1	XP_001816436.1	*Tribolium castaneum*	674	0	65%
SNMP2	Unigene6724	1217	1173	Y	1	1.72	sensory neuron membrane protein	AFI45066.1	*Dendroctonus ponderosae*	369	3E-119	49%

Supplementary TableS6 Primer used for fluorescence quantitative real-time PCR

Name	Forward primer	Reverse primer
CSP1	TGCCGACGATAAATACACCA	TCGCTGCATTTTGAACAGTC
CSP2	GCGAAGAACTGAAAAAGGTGTT	GTATTTGCCTTCTGGGTCGTAA
CSP3	CTCTTTGCGCCTTTGCTTAC	CTTCCACCAATCTGGCTTGT
CSP4	TCACGATTCACCACTCCGTA	GACGGCGTTTCAACAGAAAT
CSP5	TGCCGGTGCGTTGAGTGAAGA	TGCTCTTCTGACTCCGCCCTTGTG
CSP6	GCACTCCGGATGGACTAGAA	CCTTCTTTGGCGAACTCTTG
CSP7	GGACGACCAATACACAACCA	ATTTGCTGCAGTCCGTTTCT
CSP8	CAAAAGACGGTGACACTTCG	ACAATTTGGACGGATGGTGT
CSP9	TGAGGGCATACGTTTTAGCC	ATTGCCTTTCTCCAACATGC
CSP10	TGGTTTTGGCCGATAACAAG	TAAGGCATCAGGAAGCGTCT
CSP11	TTCGTAGGCCAGCATCAGTG	GTTTCCCGTCAGACCCCTCT
CSP12	AACGAGACGGGGCTTTTAAT	GCAAATAACGACACGCTCCT
PBP1	GCTTAGCCAGCACTGGAGTC	CAGGCGGTGTCACATACATC
PBP2	TGGTGGACATGCTTCACAAT	ATGGTCGCCTCCACATCTAC
Actin	ACATCAAGGAGAAACTCTGCTACG	CTTCATGATGGAGTTGTAGGTGGT

Supplementary TableS7 Protein number and gene accession numbers used in phylogenetic trees

OR		OBP		CSP	
**Name**	**Accession number GI**	**Name**	**Accession number GI**	**Name**	**Accession number GI**
*Dpon*OR23	471180445	*Malt*OBP6	758343053	*Tmol*CSP12	758213810
*Dpon*OR24	471180431	*Malt*OBP2	758343051	*Tmol*CSP11	758213808
*Dpon*OR25	459442396	*Acor*OBP7	729057636	*Tmol*CSP9	758213804
*Dpon*OR11	459442392	*Acor*OBP14	729057634	*Tmol*CSP8	758213802
*Dpon*OR16	459442384	*Acor*OBP3	729057632	*Tmol*CSP7	758213800
*Dpon*OR30	459442380	*Acor*OBP2	729057630	*Tmol*CSP6	758213798
*Dpon*OR45	459442372	*Dpon*OBP17	459442366	*Tmol*CSP5	758213796
*Dpon*OR7	459442368	*Dpon*OBP32	459442360	*Tmol*CSP4	758213794
*Dpon*OR19	459442362	*Dpon*OBP7	459442352	*Tmol*CSP2	758213790
*Dpon*OR43	459442346	*Ityp*OBP10	459277277	*Tmol*CSP1	758213788
*Dpon*OR40	459442342	*Ityp*OBP12	459277273	*Acorp*CSP5	807201437
*Dpon*OR6	459442334	*Ityp*OBP14	459277269	*Acorp*CSP4	807201435
*Dpon*OR14	459442332	*Ityp*OBP15	459277267	*Acorp*CSP3	807201433
*Dpon*OR33	459442328	*Ityp*OBP2	459277263	*Acorp*CSP1	807201429
*Dpon*OR39	459442318	*Ityp*OBP3	459277261	*Dpon*CSP2	471180443
*Dpon*OR3	459442312	*Ityp*OBP4	459277259	*Dpon*CSP8	471180427
*Dpon*OR5	459442306	*Ityp*OBP5	459277257	*Dpon*CSP6	471180423
*Dpon*OR36	459442300	*Ityp*OBP6	459277255	*Dpon*CSP1	471180421
*Dpon*OR37	459442298	*Ityp*OBP7	459277253	*Dpon*CSP3	471180419
*Dpon*OR27	459442292	*Hele*PBP	507144144	*Dpon*CSP4	828177651
*Dpon*OR12	459442290	*Tcas*OBP19	270006512	*Ityp*CSP1	459277247
*Dpon*OR32	459442286	*Tcas*OBP14	270006466	*Ityp*CSP4	459277243
*Dpon*OR15	459442282	*Tcas*OBP17	270006413	*Ityp*CSP5	459277241
*Ityp*OR28	459277392	*Tcas*OBP18	270006412	*Tcas*CSP20	112032008
*Ityp*OR32	459277390	*Tcas*OBP13	270006410	*Tcas*CSP19	112031988
*Ityp*OR31	459277388	*Tcas*OBP12	270006409	*Tcas*CSP18	112031961
*Ityp*OR23	459277386	*Tcas*OBP16	270006405	*Tcas*CSP17	112031943
*Ityp*OR16	459277384	*Tcas*OBPC15	270006378	*Tcas*CSP15	112031903
*Ityp*OR34	459277382	*Tcas*OBP15	270015618	*Tcas*CSP14	112031878
*Ityp*OR2	459277380	*Tcas*OBP23	270014355	*Tcas*CSP13	270011117
*Ityp*OR8	459277378	*Tcas*OBP09	270014265	*Tcas*CSP12	112031836
*Ityp*OR18	459277374	*Tcas*OBP22	270012707	*Tcas*CSP11	112031814
*Ityp*OR4	459277372	*Tcas*OBPC06	270011100	*Tcas*CSP10	112031796
*Ityp*OR13	459277370	*Tcas*OBPC02	270011097	*Tcas*CSP9	112031779
*Ityp*OR9	459277368	*Tcas*OBP10	270011094	*Tcas*CSP7	112031740
*Ityp*OR19	459277366	*Tcas*OBP4	270009294	*Tcas*CSP6	112031719
*Ityp*OR27	459277364	*Tcas*OBP11	270009247	*Tcas*CSP5	112031698
*Ityp*OR43	459277362	*Tcas*OBP2	270009228	*Tcas*CSP4	112031683
*Ityp*OR36	459277360	*Tcas*OBP07	270008145	*Tcas*CSP2	112031657
*Ityp*OR6	459277358	*Tcas*OBP24	270008128	*Dmel*CSP98a	665395309

*Ityp*OR35	459277356	*Tcas*OBPC20	270004977	*Dmel*CSP93b	78711852
*Ityp*OR1	459277354	*Tcas*OBPC18	270002750	*Dmel*CSP75a	78707583
*Tcas*OR245	270016239	*Tcas*OBPC11	270001292	*Dmel*CSP83c	78707549
*Tcas*OR146	270008285	*Dmel*OBP28a	17647793	*Dmel*CSP7a	78707537
*Tcas*OR35	270002961	*Dmel*OBP99c	24651098	*Dmel*CSP56a	78707262
*Tcas*OR29	270002957	*Dmel*OBP44a	19921806	*Dmel*CSP46a	78707184
*Tcas*OR325	270002902	*Dmel*OBP69a	24663268	*Dmel*CSP86a	78706742
*Tcas*OR28	270002794	*Dmel*OBP84a	17136918	*Dmel*CSP84a	78706694
*Tcas*OR37	270002782	*Dmel*OBP19c	24643505	*Dmel*CSP74a	78706694
*Tcas*OR205	270001337	*Dmel*OBP56c	386768340	*Dmel*CSP53a	62471725
*Tcas*OR186	270001307	*Dmel*OBP57a	24656247	*Dmel*CSP42a	45552485
*Tcas*OR66	270001280	*Dmel*OBP49a	24653178	*Dmel*CSP38a	45552427
*Tcas*OR204	270001276	*Dmel*OBP8a	24640769	*Dmel*CSP29a	28574075
*Tcas*OR227	270004963	*Dmel*OBP50b	24653633	*Dmel*CSP87a	24646535
*Tcas*OR9a	642937146	*Dmel*OBP51a	24653777	*Bmor*CSP1	112032026
*Tcas*Orco	642925912	*Dmel*OBP83ef	24644507	*Bmor*CSP2	112032057
*Tcas*OR92a	642920568	*Dmel*OBP59a	28573615	*Bmor*CSP3	145694413
*Tcas*OR2a	642912833	*Dmel*OBP50a	24653631	*Bmor*CSP4	112032114
*Amel*OR37	339715145	*Dmel*OBP93a	24648633	*Bmor*CSP5	112032133
*Amel*OR30	339715140	*Dmel*OBP85a	45550714	*Bmor*CSP6	112032153
*Amel*OR27	339715138	*Dmel*OBP47b	45550714	*Bmor*CSP7	112032179
*Amel*OR26	339715136	*Dmel*OBP73a	161084444	*Bmor*CSP8	112032195
*Amel*OR25	339715134	*Dmel*OBP58d	24658441	*Bmor*CSP9	112032214
*Amel*OR19	339715130	*Bmor*OBP012	261245103	*Bmor*CSP10	112983056
*Amel*OR2	201023349	*Bmor*OBPC17	255652863	*Bmor*CSP11	112032244
*Amel*OR115	339715185	*Bmor*OBP497	237648976	*Bmor*CSP13	112032283
*Amel*OR105	339715179	*Bmor*OBP495	237648972	*Bmor*CSP14	112032298
*Amel*OR57	339715164	*Bmor*OBP013	261245105	*Bmor*CSP15	112032318
*Amel*OR53	339715157	*Bmor*OBP496	237648974	*Bmor*CSP16	112032338
*Amel*OR50	339715151	*Bmor*OBP7	226531207	*Amel*CSP3_p	58585106
*Amel*OR41	339715147	*Bmor*OBP3	226501560	*Amel*CSP1_p	118150502
*Amel*OR35	339715142	*Bmor*OBP6	226501798	*Amel*CSP6_p	118150500
*Amel*OR55	339715159	*Bmor*OBP5	226501720	*Amel*CSP4_p	118150492
*Amel*OR56	339715162	*Bmor*OBP2	226501484	*Amel*CSP4_p	112031616
*Amel*OR51	339715153	*Bmor*GOBP2	1155064	*Amel*CSP6	112031635
*Dmel*Orco	24644231	*Bmor*GOBP1	1155062	*Amel*CSP4	112031599
*Dmel*OR42a	22023972	*Bmor*PBP1	1155066	*Amel*CSP3	112031581
*Dmel*OR43b	22023986	*Bmor*PBP3	133919126	*Amel*CSP2	112031564
*Dmel*OR47b	45549172	*Bmor*PBP2	133919124	*Amel*CSP1	112031547
*Dmel*OR23a	28574001	*Amel*ASP4	18140739	*Amel*CSP2_p	118150486
*Dmel*OR10a	17986023	*Amel*ASP2	18140741		
*Dmel*OR35a	24584456	*Amel*ASP6	18140747		
*Dmel*OR2a	17933522	*Amel*ASP5	18140749		
*Dmel*OR67b	24661763	*Amel*ASP1	18140743		

GR	
**Name**	**Accession number GI**
*Dpon*GR1	459442338

*Dmel*OR59c	17647785	*Amel*OBP5	58585124	*Dpon*GR3	459442296
*Dmel*OR7a	17530847	*Amel*OBP4	58585118	*Ityp*GR6	459277307
*Dmel*OR33c	17647767	*Amel*OBP19	94158731	*Ityp*GR4	459277305
*Dmel*OR82a	24643875	*Amel*OBP2	58585122	*Ityp*GR3	459277303
*Dmel*OR98a	24650735	*Amel*OBP14	94158822	*Tcas*GRa	221136983
*Dmel*OR19a	24643445	*Amel*OBP11	94158842	*Tcas*GRb	163716784
*Dmel*OR65a	24659319	*Amel*OBP7	94158720	*Tcas*GRc	163716754
*Dmel*OR88a	24646756	*Amel*OBP21	94158718	*Tcas*GR178	270006491
*Dmel*OR92a	24648414	*Amel*OBP17	94158711	*Tcas*GR97	270006483
*Dmel*OR9a	17986019	*Amel*OBP16	94158709	*Tcas*GR2	270006476
*Dmel*OR56a	24655965	*Amel*OBP10	94158674	*Tcas*GR26	270015820
*Dmel*OR49a	28573387	*Amel*OBP 1	58585128	*Tcas*GR27	270015819
*Dmel*OR85d	17986171	*Amel*OBP 6	58585126	*Tcas*GR168	270011186
*Dmel*OR45a	28573313	*Amel*OBP12	94158820	*Tcas*GR154	270011184
*Dmel*OR74a	17986153	*Amel*OBP20	94158813	*Tcas*GR153	270011183
*Dmel*OR22c	24580998	*Amel*OBP18	94158830	*Tcas*GR125	270011173
*Dmel*OR1a	24638847	*Amel*OBP3	94158830	*Tcas*GR118	270011167
*Dmel*OR30a	24582997	*Amel*OBP15	94158668	*Tcas*GR117	270011166
*Dmel*OR13a	24642365			*Tcas*GR1	270011146
*Dmel*OR94a	17738133	**IR**		*Tcas*GR24	270009311
*Dmel*OR24a	221472522	**Name**	**Accession number GI**	*Tcas*GR166	270008279
*Dmel*OR83a	24644225	*Tmol*IR6	758213862	*Tcas*GR18	270008276
*Bmor*OR18	290651022	*Tmol*IR4a	758213858	*Tcas*GR14	270008272
*Bmor*OR36	290650661	*Tmol*IR4b	758213858	*Tcas*GR13	270008271
*Bmor*OR16	290566751	*Tmol*IR2	758213854	*Tcas*GR12	270008270
*Bmor*OR11	290563295	*Tmol*IR1	758213852	*Tcas*GR11	270008269
*Bmor*OR54	290560855	*Dpon*IR8a	471180437	*Tcas*GR10	270008268
*Bmor*OR59	290560851	*Dpon*IR56e.1	459442394	*Tcas*GR7	270008265
*Bmor*OR14	290560836	*Dpon*IR75x	459442388	*Tcas*GR5	270008263
*Bmor*OR55	290559929	*Dpon*IR76b	459442376	*Tcas*GR3	270008261
*Bmor*OR57	261245111	*Dpon*IR75p.1	459442364	*Tcas*GR164	270002945
*Bmor*OR17	254939545	*Dpon*IR75p.1	459442354	*Tcas*GR87	270002937
*Bmor*OR8	254939543	*Dpon*IR93a.1	459442350	*Tcas*GR82	224458358
*Bmor*OR60	240255406	*Dpon*IR21a.1	459442326	*Amel*GR7	571550487
*Bmor*OR9	182509188	*Dpon*IR93a.2	459442324	*Amel*GR10	339715206
*Bmor*OR25	162462502	*Dpon*IR75q	459442322	*Amel*GR32	571550434
*Bmor*OR37	162461284	*Dpon*IR25a	459442302	*Amel*GR64f	571529490
*Bmor*OR45	162461258	*Ityp*IR25a	459277285	*Amel*GR43a	328778564
*Bmor*OR33	158711751	*Dmel*IR25a	316994955	*Dmel*GR43a	47117920
*Bmor*OR35	158508574	*Dmel*IR10a	158031786	*Dmel*GR61a	17986119
*Bmor*OR15	148298756	*Dmel*IR52c	21645383	*Dmel*GR64a	24657115
*Bmor*OR30	148298744	*Dmel*IR56a	21626963	*Dmel*GR21a	118500892
*Bmor*OR2	112983084	*Dmel*IR64a	7295466	*Dmel*GR5a	24639922
*Bmor*OR4	112982926	*Dmel*IR67a	442631348	*Dmel*GR66a	281365837

*Bmor*OR10	162462631	*Dmel*IR41a	442622278	*Dmel*GR63a	221330835
*Bmor*OR7	163838688	*Dmel*IR84a	442617872	*Dmel*GR32a	45549158
*Bmor*OR47	162462595	*Dmel*IR11a	442616103	*Dmel*GR64f	45551511
*Bmor*OR21	162462571	*Dmel*IR7b	442615429	*Dmel*GR68a	24662881
*Bmor*OR12	162462524	*Dmel*IR75d	386771401	*Dmel*GR33a	45549284
*Bmor*OR34	158711753	*Dmel*IR94e	221458656	*Dmel*GR93a	24648814
*Bmor*OR38	158508576	*Dmel*IR54a	221330374	*Dmel*GR57a	17986103
*Bmor*OR41	148298822	*Dmel*IR52a	221330289	*Dmel*GR28a	45549155
*Bmor*OR42	148298766	*Dmel*IR7f	221329768	*Dmel*GR10a	24641287
*Bmor*OR19	148298665	*Dmel*IR21a	161076594	*Dmel*GR8a	28571153
*Bmor*OR1	112983558	*Dmel*IR62a	85725098	*Dmel*GR59f	28573623
*Bmor*OR6	112982988	*Dmel*IR100a	45550870	*Dmel*GR39b	24585658
*Bmor*OR3	112982950	*Dmel*IR60a	24762594	*Dmel*GR94a	24649189
*Bmor*OR5	112982948	*Dmel*IR68b	24663135	*Dmel*GR58c	28573606
*Bmor*OR29	290650771	*Dmel*IR56d	24655848	*Dmel*GR36a	45550994
*Bmor*OR27	290650676	*Dmel*IR51b	24653793	*Dmel*GR22a	24580943
*Bmor*OR63	290563364	*Dmel*IR48b	24652806	*Dmel*GR77a	24667642
*Bmor*OR56	290563360	*Dmel*IR47a	24652423	*Dmel*GR47b	45551079
*Bmor*OR20	290563309	*Dmel*IR85a	24645175	*Dmel*GR89a	45550757
*Bmor*OR61	290560861	*Dmel*IR20a	24643741	*Dmel*GR98d	24650640
*Bmor*OR22	290560841	*Dmel*IR7a	24640399	*Dmel*GR92a	45551934
*Bmor*OR13	290559921	*Dmel*IR84a	316994959	*Dmel*GR85a	24645557
*Bmor*OR24	240255412	*Dmel*IR76b	316994957	*Dmel*GR10b	17986021
*Bmor*OR46	240255410	*Dmel*IR8a	316994953	*Dmel*GR9a	24640947
*Bmor*OR49	197322776	*Dmel*IR64a	316994961	*Bmor*GR68	350536295
TmolOrco	no	*Dmel*IR75a	312434883	*Bmor*GR67	350536275
MacrOrco	no	*Dmel*IR93a	440217690	*Bmor*GR60	195963349
DponOrco	332373962	*Dmel*IR92a	440217656	*Bmor*GR45	195963347
AcorOrco	KM251654	*Dmel*IR87a	440217387	*Bmor*GR9	195963345
				*Bmor*GR8	195963343
				*Bmor*GR10	912719236

**PDE**	
**Name**	**Accession number GI**
*Sinf*ODE-CXE28	669296908
*Sinf*ODE-CXE26	669296906
*Sinf*ODE-CXE20	669296904
*Sinf*ODE-CXE19	669296902
*Sinf*ODE-CXE18	669296900
*Sinf*ODE-CXE16	669296898
*Sinf*ODE-CXE14	669296896
*Sinf*ODE-CXE13	669296894
*Sinf*ODE-CXE12	669296892
*Sinf*ODE-CXE11	669296890
*Sinf*ODE-CXE10	669296888
*Sinf*ODE-CXE9	669296886

**SNMP**	
**Name**	**Accession number GI**
*Tmol*SNMP2	758213866
*Tmol*SNMP1	758213864
*Dpon*SNMP2	471180467
*Dpon*SNMP1a	471180441
*Ityp*SNMP1	459277281
*Ityp*SNMP2	459277279
*Tcas*SNMP1	189236600
*Tcas*SNMP2	91092044
*Dmel*SNMP1	440217709
*Dmel*SNMP2	158516729

*Sinf*ODE-CXE6	669296884		HarmSNMP1	27462830
*Sinf*ODE-CXE5	669296882		*Bmor*SNMP1	112984488
*Sinf*ODE-CXE3	669296880		*Bmor*SNMP2	827550980
*Sinf*ODE-CXE1	669296876		*Amel*SNMP2	374253753
ApolODE	29465750		*Amel*SNMP1	384872681
PdivPDE	47779228			
PjapPDE	62002227			
